# Ethnobotanical study of medicinal plants used by the Yi people in Mile, Yunnan, China

**DOI:** 10.1186/s13002-024-00656-1

**Published:** 2024-02-23

**Authors:** Hongrui Li, Caiwen Huang, Yanhong Li, Pujing Wang, Jingxian Sun, Zizhen Bi, Shisheng Xia, Yong Xiong, Xishan Bai, Xiangzhong Huang

**Affiliations:** 1 School of Ethnology and History, Yunnan Minzu University, Kunming, 650504 China; 2 Key Laboratory of Chemistry in Ethnic Medicinal Resources, State Ethnic Affairs Commission & Ministry of Education, Yunnan Minzu University, Kunming, 650504 China

**Keywords:** Yi people, Ethnobotany, Traditional medicine, Traditional knowledge, Mile City

## Abstract

**Background:**

The Yi people are a sociolinguistic group living in Mile City, which is their typical settlement in southeastern Yunnan, China. Over the long history of using medicinal plants, the Yi people have accumulated and developed a wealth of traditional medicinal knowledge, which has played a vital role in their health care. However, only a few studies have been performed to systematically document the medicinal plants commonly used by the Yi people. This study provides fundamental data for the development and application of ethnomedicine as well as supports the conservation of the traditional medical knowledge of the Yi people.

**Methods:**

This study was conducted from May 2020 to August 2022 and involved five townships in Mile. Information regarding medicinal plants was obtained through semistructured interviews, key informant interviews, and participatory observation. The collected voucher specimens were identified using the botanical taxonomy method and deposited in the herbarium. Ethnobotanical data were analyzed using informant consensus factor, relative frequency of citation, and fidelity level.

**Results:**

In total, 114 informants distributed in five townships of Mile were interviewed. The Yi people used 267 medicinal plant species belonging to 232 genera and 104 families to treat various diseases. Asteraceae, Lamiaceae, and Fabaceae were the most commonly used plant families by the Yi people. In addition, herbs were most commonly used by the Yi people. Whole plants and roots were the preferred medicinal parts. Decoctions were the most common method of herbal medicine preparation. There are 49 different recorded diseases treated by Yi medicinal plants, and among them, respiratory diseases, rheumatism, traumatic injury, fractures, and digestive system diseases have the largest number of species used. A quantitative analysis demonstrated that plants such as *Zingiber officinale*, *Lycopodium japonicum*, *Aconitum carmichaelii*, *Panax notoginseng*, *Cyathula officinalis*, and *Leonurus japonicus* played crucial roles in disease prevention and treatment.

**Conclusion:**

Traditional knowledge of medicinal plants is closely associated with the social culture of the local Yi people. The medicinal plants used for health care in the study area were diverse. Local healers were skilled at using medicinal plants to treat various diseases. Their treatment methods were convenient and unique, exhibiting distinctive regional characteristics. However, the inheritance of their traditional medicinal knowledge and protection of wild medicinal plant resources are facing serious challenges, including the decreasing number of local healers, aging of healers, lack of successors, and excessive harvesting of medicinal plant resources. This ethnobotanical survey provides a useful reference for the sustainable utilization and protection of medicinal plant resources in Mile and the inheritance of traditional medicinal knowledge of the Yi people.

## Background

Since ancient times, medicinal plants have been widely used in healthcare systems to treat various diseases [1]. The World Health Organization Traditional Medicine Strategy 2014–2023 indicated that traditional remedies, practitioners, and herbs provide health care for millions of people [2]. It is estimated that approximately 80% of the global population still depends on traditional medicine for primary health care [3], especially those living in remote mountainous areas. Various forms of traditional medicine are practiced throughout the world, such as Indian medicine, Arabic medicine, and Chinese medicine. A strong connection exists between traditional Chinese medicine and Arabic and Indian medicine, all of which play an integral role in spreading the human medicinal civilization [4]. In China, ethnic traditional medicine is an inseparable part of traditional Chinese medicine. As a multiethnic country, there are 55 ethnic minorities in China, each with their own unique traditional medicine [5]. For instance, the Shui people are experienced in treating bone fractures, traumatic injuries, and snake bites, which are their occupational hazards [6], and the Yao people have accumulated rich experience in treating skin diseases and rheumatism using medicinal baths [7]. These ethnomedicine systems have developed from a wide range of healthcare systems, experiences, and beliefs, possessing distinct ethnic and regional medical characteristics [8]. Therefore, the Chinese government has introduced several policies for the development and protection of traditional medicine. For instance, in 1951, the *Ethnic Minorities Health Work Plan of China* recommended that native doctors who cure diseases using traditional medicinal plants should be united and supported. During the 1960s and 1970s, the Chinese government established a primary healthcare system, known as “barefoot doctors,” to provide health care to rural residents who have limited access to medical services [9]. These barefoot doctors were familiar with traditional medicinal plants and experienced in using them to treat diseases. Between 2011 and 2020, the Chinese government implemented the *Fourth National Survey of Chinese Materia Medica Resources* to improve the management of traditional medicinal resources.

In China, the Yi people are the sixth largest ethnic minority with a population of approximately 9.83 million. Approximately 61% of the Yi people reside in Yunnan Province, and their population is also distributed throughout Guizhou, Sichuan, and Guangxi Provinces. The Yi people speak their indigenous language, which belongs to the Tibetan–Burman language family within the Sino–Tibetan family. They developed their medical knowledge system during the long struggle against disease and harsh environments, and the rise of Yi medicine can be traced back to the Eastern Han Dynasty, 1800 years ago [10]. Traditional medical knowledge was recorded and summarized with the ancient Yi script and compiled into specialized books. To date, some books related to Yi medicine have been published, such as “*Shuangbai Yi Medicine Book*,” “*Qigu shu*,” “*Materia Medica in South Yunnan*,” “*Ailao Materia Medica*,” “*The Theory and Application of Yi Medicine*,” and “*Yi Medicine Records*.” Among them, “*Shuangbai Yi Medicine Book*” is a crucial Yi medicine book published in 1566, which is the oldest publication completely recording Yi medicine. This book collected a large amount of information concerning medicinal plants from the local people [11]. Thousands of medicinal plants were recorded in these books, which were valuable references in clinics.

Mile is a county-level city with multiethnic communities located in the south of Yunnan Province, China, with a high diversity of species and a well-covered forest. The Han, Yi, and Dai people are the major indigenous ethnic groups in Mile. According to the sixth census, the Yi population is > 165,000, amounting for approximately 30.72% of the total population of the city, and most of them live in mountainous regions with complex terrain and poor transportation. Historically, most locals depended on mountain agriculture to sustain a self-sufficient economic system, and communication with other ethnic groups was limited. Consequently, the native culture and customs of the Yi people were less influenced by other ethnic cultures, and the traditional knowledge of medicinal plants was relatively well preserved. The local Yi people strongly believed in animism and nature worship, which played a significant role in maintaining a harmonious relationship between man and nature [12]. Over time, to adapt to the local environment, the local Yi people accumulated extensive knowledge of medicinal plants, which contributed to their survival and community flourishing.

Over the past few decades, the demand for complementary and alternative medicine and traditional medicine has dramatically increased. Traditional medicinal plants were not only used as regional and traditional treatments but also registered as official medicines and certified by the Pharmacopeia. In recent years, several products based on Yi medicines have been exploited and reported to provide positive social and financial benefits. For instance, *Yunnan Baiyao*, *the Capsule of Paiduyangyan*, *the Injection of Yunnan Dengzhanhua*, and the *Capsule of Yixinkang* were developed based on ancient Yi medicine culture and folk medicines [13]. Nevertheless, the increased demand for medicinal plants has resulted in the overexploitation of some medicinal plant resources. Therefore, it is necessary to protect the biodiversity and use resources sustainably. Meanwhile, the traditional knowledge and culture related to medicinal plant resources should also be protected and inherited.

Although several ethnobotanical investigations have been conducted, ethnobotanical investigation of Yi medicinal plants is still at an early stage. Currently, some traditional knowledge held by the Yi people in Mile has not been scientifically documented and is at risk of disappearing. Therefore, it is extremely urgent to preserve the traditional knowledge regarding herbal medicines in Mile. Accordingly, this ethnobotanical survey was conducted with the following purposes: (1) to document commonly used medicinal plants and associated traditional knowledge, (2) to analyze the characteristics and utilization of medicinal plants, and (3) to provide useful information for promoting the development and application of ethnomedicine as well as for supporting the conservation of Yi traditional medical knowledge.

## Methods

### Study area

**Fig. 1:**
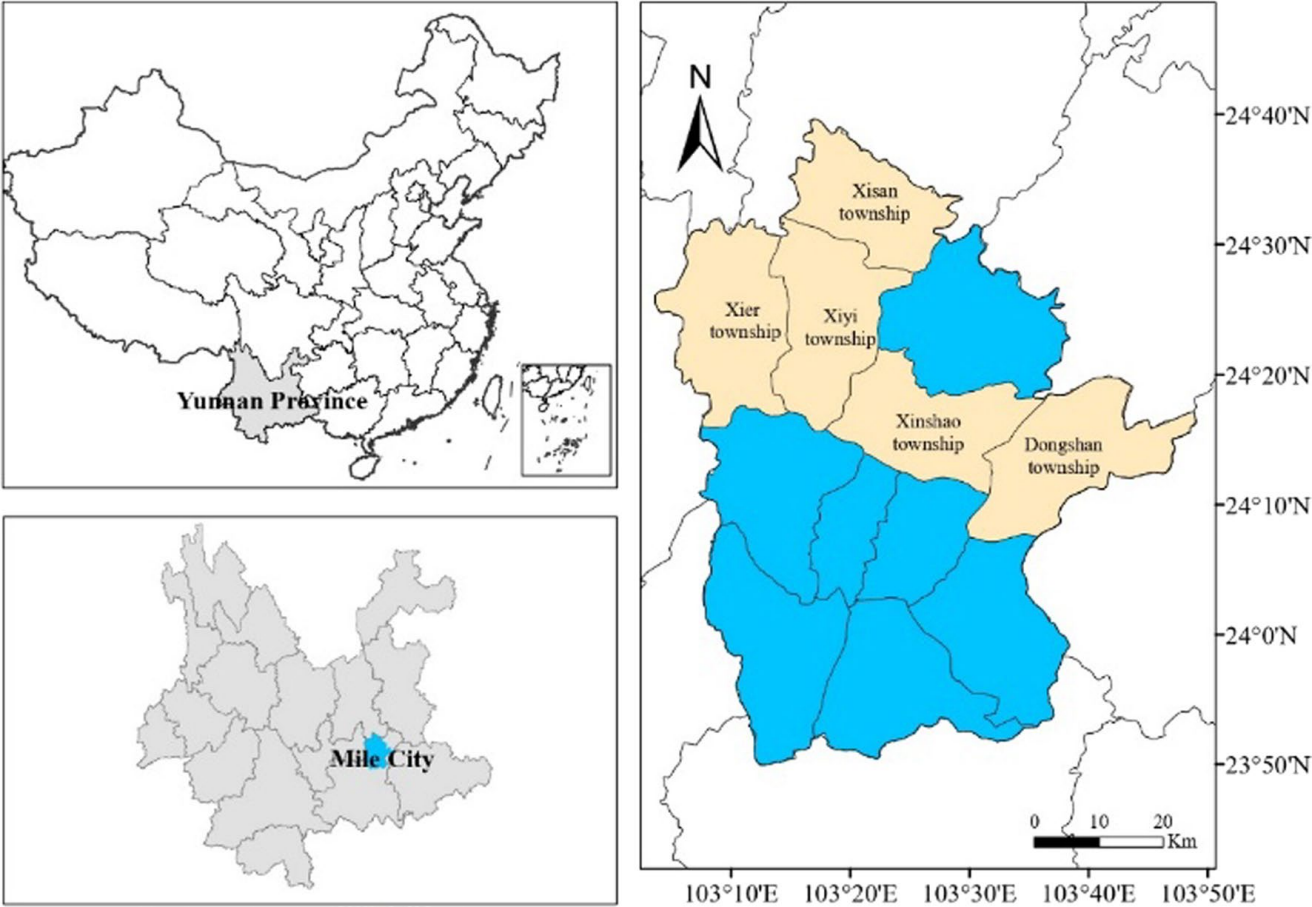
Sketch map of the study area

Mile (103° 04′–103° 49′ E and 23° 50′–24° 39′ N) is a county-level city located in the southeast Yunnan Province, China (Fig. 1). It is known as the north gate of Honghe Hani and Yi Autonomous Prefecture, with Kunming to the north, Kaiyuan to the south, Wenshan to the east, and Yuxi to the west. Mile is composed of 12 townships and covers an area of approximately 4004 km^2^, with an east–west distance of approximately 78 km and a north–south span of approximately 50 km [14]. The lowest point is at an altitude of approximately 862 m, and the highest point is at an altitude of approximately 2315 m. Mile is located on the Yunnan–Guizhou Plateau, a typical karst landform area, where limestone is widely distributed. The climate of this area is dominated by a subtropical monsoon climate, with a mean annual temperature of 18.8 °C and a mean annual rainfall of 835.4 mm. The unique climate and geological environment of this area provide suitable habitats for various flora and fauna.

### The Yi people

Most of the Yi communities in Mile are distributed in mountainous areas. They are primarily engaged in mountain agriculture, including the cultivation of tobacco, walnuts, corn, wheat, beans, fruits, medicinal herbs, and vegetables. Traditional houses of the Yi people are Tuzhangfang, which is an earthenly built wooden-structured house with two stories. The kitchen, living room, and cattle barn were arranged on the first floor, and the bedrooms and storage were arranged on the second floor. However, with the development of the social economy in recent decades, the Yi people’s houses have been converted into modern cement buildings. The local Yi people often cultivate landscape plants and commonly used medicinal plants around their homes.

The Yi language is widely spoken in Yi communities. However, almost all Yi people in the study area are aware of both Yi and Chinese languages because they often communicate with both the Yi and Han people during their daily lives. With the continuous improvement of transportation facilities, travel for people living in mountainous areas is becoming increasingly convenient. Therefore, the Yi people interact with other cultures more frequently and widely than ever before. Meanwhile, the local Yi people have learned valuable information concerning medicinal plants through interaction and trading herbs with others.

The Yi people in the study area strongly believed in animism and nature worship. They worshiped various natural objects, including water, fire, trees, and tigers, which are closely related to their daily lives. To date, a variety of worship customs have been passed down in the study area, such as “Dragon sacrifice,” “Torch festival,” “Mizhi festival,” and “Tiger sacrifice,” all of which play an essential role in traditional Yi folk culture [15]. Nature worship has important social and cultural significance, which can promote the development of farming civilization, ecological consciousness, art, and culture in the Yi area. Bimoism is the indigenous religion of the Yi people. In Yi culture, Bimo is a priest who performs religious rituals and treats diseases in Yi communities. As an inheritor of Yi culture, Bimo is a master of the Yi language, philosophy, history, astronomy, folklore, ethics, art, medicine, agriculture, and so on. The Bimo Sutras composed by Bimo have recorded rich traditional Yi medicinal knowledge, which is an important reference for investigating Yi medicine.

### Data collection

**Fig. 2:**
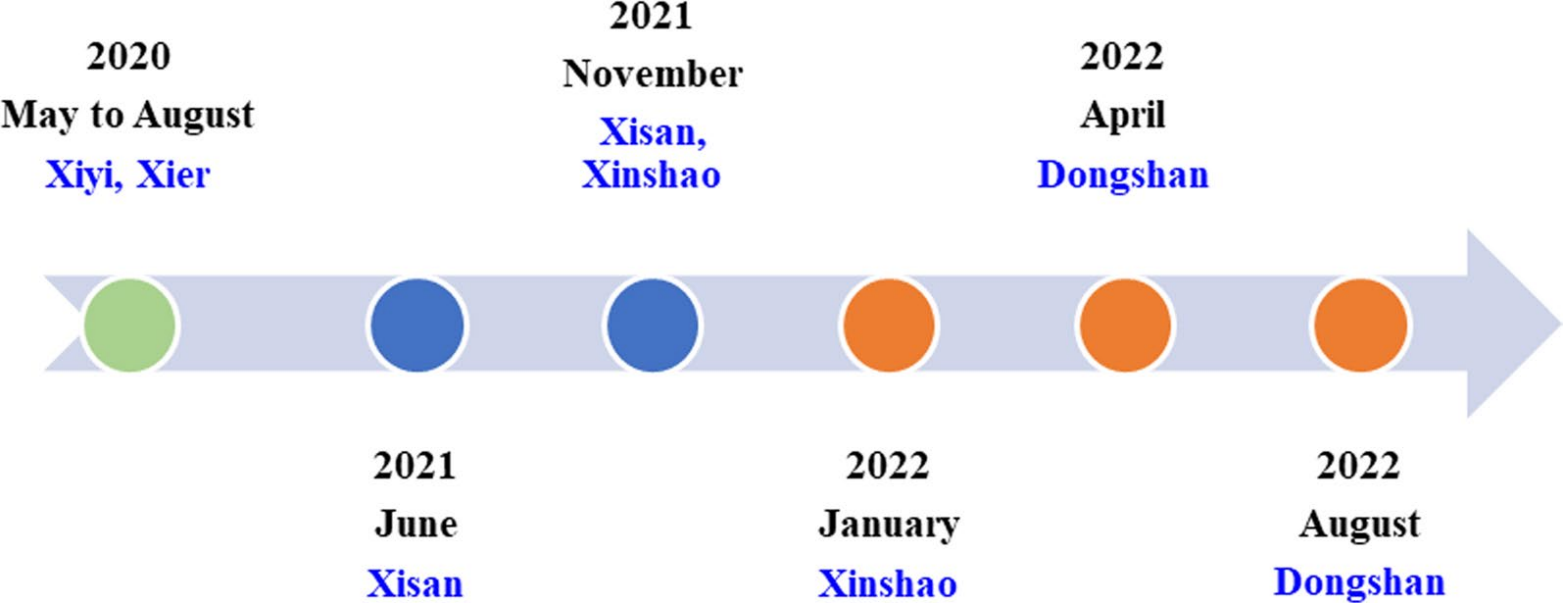
Field surveys performed from May 2020 to August 2022

**Fig. 3:**
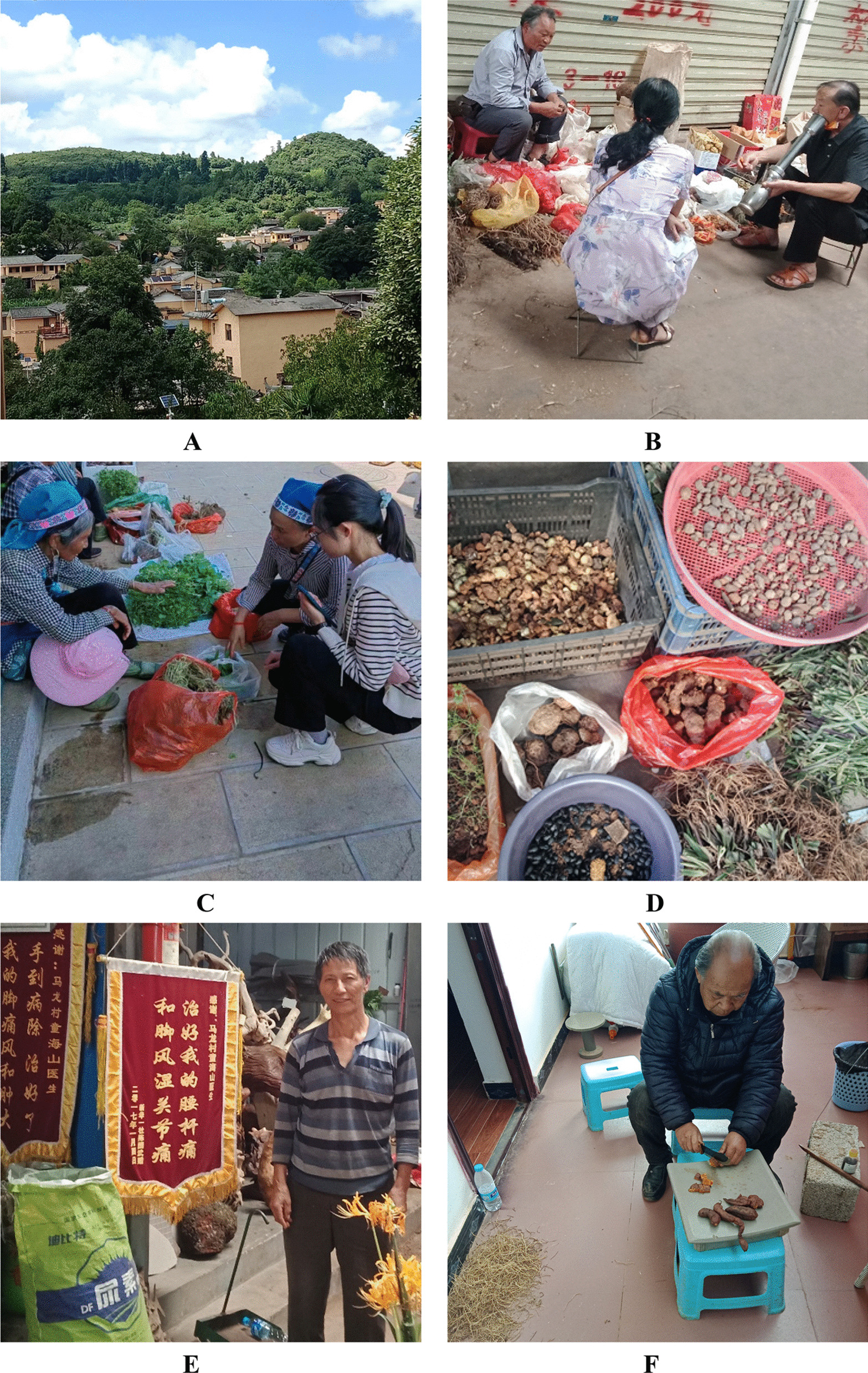
**A** Yi village and the surrounding farming fields; **B**–**D** Collecting information from herb trading markets (**B** Mile market; **C** and **D** Huakou market in Xisan township); **E** Local Yi healer; **F** A male Yi folk doctor is preparing medicine at home. (The photos were taken by the author H.L. and P.W., Photos **A–E** were taken in June 2021, and Photo **F** was taken in January 2022.)

Six field surveys were conducted from May 2020 to August 2022 to collect ethnobotanical data in five townships (Xier, Xiyi, Xisan, Xinshao, and Dongshan) (Fig. 2). Traditional ethnobotanical knowledge was collected from 114 informants (74 men and 40 women) through semistructured interviews, key informant interviews, and participatory observations (Fig. 3). In the study area, most individuals are familiar with herbal medicine to some extent. However, only a few people are recognized as experienced healers. Snowball sampling was used to select the key informants based on the recommendations of local people. A total of 46 key informants (25 men and 21 women) were selected, who were healers and vendors with relatively extensive medical experience. All informants were local residents and aged between 27 and 86 years. Two coauthors (Zizhen Bi and Shisheng Xia) of this article are local Yi healers whose native language is Yi. Therefore, communication with the local people was very smooth during the field investigation. This study was conducted according to the International Society of Ethnobiology Code of Ethics [16] and the American Anthropological Association Code of Ethics [17]. Consent was obtained from the informants before field investigations. Specimens were collected from the field of investigation with the assistance of local guides, and voucher specimens were identified by comparison with the *Flora of China*, *Flora of Yunnan*, and botanical websites (http://www.iplant.cn/, https://www.worldfloraonline.org/). The obtained information was cross-checked with that of other informants.

### Data analysis

Statistical analysis was conducted using Microsoft Office Excel. Informant consensus factor (ICF) was used to analyze the variation in medicinal plant species used by different healers to treat a particular disease category [18]. It was calculated using the following formula: ICF = (Nur − Nt)/(Nur − 1), where Nur is the total number of plant species used by all informants to treat a particular disease category, and Nt is the number of plant species commonly used by all informants to treat this disease category [19].

Relative frequency of citation (RFC) was used to evaluate the importance of plants used by local healers to treat various diseases [20]; it was calculated as follows: RFC = FC/N, where FC is the number of prescriptions mentioning the use of a plant species, and N is the total number of prescriptions in the survey.

Fidelity level (FL) was used to evaluate the significance of a species for a given purpose [21]; it was calculated using the following formula: FL (%) = (Ip/Iu) × 100, where Ip is the number of informants who suggested the use of a species for the same major purpose, and Iu is the total number of informants who mentioned the use of the species for any purpose [22].

## Results and discussion

### Demographics of the informants

Details of the informants were obtained through face-to-face interviews. Table 1 shows the demographic information of the informants. The 114 informants were distributed in the following five townships: Xiyi (17), Xier (13), Xisan (38), Xinshao (21), and Dongshan (25). The majority of informants were local farmers (44.74%), followed by healers (35.09%) and vendors (20.18%), with the latter two being familiar with herbal medicines. There were fewer female informants than male informants, with 40 (35.09%) being women and 74 (64.91%) being men; this could have been possibly due to the conservative succession manner of traditional medicinal knowledge in the Yi community. The local Yi healers prefer teaching traditional medicine to male members rather than female members. This mode of transmission is very different from that of the Yi people in the Xiaoliangshan region. It was reported that the traditional Yi medicine had often been passed down and used as an important source of healing for families and tribes in Xiaoliangshan, and all members of a family and tribe can learn medical knowledge without the limitation of gender [23]. The traditional division of labor in the study area could be another possible explanation for this result. In the study area, women were primarily responsible for domestic duties and farming, whereas men were primarily responsible for technical work. Consequently, men have more opportunities than women to acquire traditional medicinal knowledge.

**Table 1:** Detailed demographic information about the informants in the study area

Factors	Categories	Number of people	Proportion (%)
Township	Xiyi	17	14.91
	Xier	13	11.40
	Xisan	38	33.33
	Xinshao	21	18.42
	Dongshan	25	21.93
Vocation	Healer	40	35.09
	Vendor	23	20.18
	Farmer	51	44.74
Gender	Male	74	64.91
	Female	40	35.09
Age	Less than 30	2	1.75
	30–40	6	5.26
	41–50	16	14.04
	51–60	33	28.95
	61–70	42	36.84
	More than 70	15	13.16
Education	None	31	27.19
	Primary	53	46.49
	Secondary	26	22.81
	Tertiary	4	3.51
Ways to acquire medical knowledge	Learned by self	40	35.09
	Learned from parents	59	51.75
	Learned from teacher or master	15	13.16

In the study area, most informants were not educated or trained in formal institutions. Primary and secondary education were the predominant educational levels (Table 1). Regarding medicinal knowledge, most of it was inherited from family members (51.75%), learned by self-study (35.09%), or taught by masters and teachers (13.16%). In this study, the oldest male healer from Zheyi village in Xisan township was 86 years old and had been practicing healing for approximately 60 years. The youngest healer, a pharmacy undergraduate, was 27 years old and had been practicing treatment for 2 years. As shown in Table 1, among all the informants who were familiar with medicinal plants, the majority were aged 41–70 years (*n* = 91, 79.82%), whereas 15 informants were aged > 70 years, and only 8 informants were aged < 41 years, which is similar to that in other regions [24, 25]. Traditional medicinal knowledge was primarily held by the local older generations who had more trust in traditional medicine practice than the younger generations. Therefore, the preservation, inheritance, popularization, and application of traditional Yi medicine were facing a significant threat because it would be lost with the death of the older generations.

Among the local healers interviewed in this study, only four healers had their sons as successors, whereas the remaining 36 healers were still without successors. This could be attributed to the following two major factors: (1) several young people are unwilling to learn traditional Yi medicinal knowledge because they believe that it is outdated, boring, and useless, whereas traditional Chinese medicine and Western medicine are more widely accepted [26] and (2) learning Yi traditional medicine requires considerable effort and a large amount of time and yields only a meager income; hence, young people prefer working in other fields for higher incomes.

To enable a more diverse demographic of the Yi people in traditional medicinal knowledge and promote the inheritance and development of Yi medicine, the local government could organize a series of training sessions or encourage cooperation between the Yi community and medical colleges and universities in Yunnan.

### Diversity of medicinal plants used in the study area

This study recorded 267 medicinal plant species used by local people for treating various diseases. These plants were distributed in 104 families and 232 genera. Table 2 shows the information of each plant species, including the voucher number, scientific name, family name, Chinese name, vernacular name, habit, medicinal part, preparation method, administration form, and therapeutic uses. The voucher specimens were prepared and deposited in the herbarium of the Key Laboratory of Chemistry in Ethnic Medicinal Resources, State Ethnic Affairs Commission & Ministry of Education, Yunnan Minzu University, Kunming, China.

**Table 2:** Inventory of medicinal plants traditionally used by Yi people in Mile

Voucher number	Scientific name	Family	Chinese name	Vernacular name	Habit	Medicinal part	Preparation method	Administration form	Therapeutic uses	FC	RFC
HXZ-41001-4	*Sambucus adnata *Wall. ex DC	Viburnaceae	Xuemancao	Zimenianmezai	H	Whole plant	D	O	Irregular menstruation, rheumatism	2	0.010
HXZ-70625-2	*Zanthoxylum nitidum* DC	Rutaceae	Liangmianzhen	Shanbazai	WV	Root	D	EW	Snake bite, stomachache, toothache, traumatic injury	3	0.015
HXZ-50419-3	*Salvia miltiorrhiza* Bunge	Lamiaceae	Zidanshen	Binlangsizai	H	Root	D	O	Irregular menstruation, rheumatism, traumatic injury	4	0.020
HXZ-41001-14	*Achyranthes aspera* L	Amaranthaceae	Tuniuxi	Niansizai	H	Root	D	O	Pharyngitis, rheumatism, traumatic injury	3	0.015
HXZ-41002-22	*Rubus parvifolius* L	Rosaceae	Maomei	Sannianzai	S	Leaf	D	O	Cold, dysmenorrhea, scabies, stone	1	0.005
HXZ-41002-29	*Reynoutria multiflora* (Thunb.) Moldenke	Polygonaceae	Heshouwu	Anaigumezai	HV	Root tuber	D	O	Constipation, lumbosacral pain, metrorrhagia	4	0.020
HXZ-41003-11	*Solanum violaceum* Blume	Solanaceae	Citianqie	Mengpushanlaisongzai	H	Fruit	PU	EA	Toothache	1	0.005
HXZ-4103-14	*Pistacia chinensis* Bunge	Anacardiaceae	Huanglianmu	Longmianzai	T	Leaf	D	O	Dysentery	1	0.005
HXZ-41003-15	*Corallodiscus flabellatus* (Craib) B.L.Burtt	Gesneriaceae	Shidancao	Luomebusezai	H	Whole plant	D	O	Pharyngitis, rheumatism, traumatic injury	2	0.010
HXZ-41004-3	*Micromeria biflora* Benth	Lamiaceae	Jiangweicao	Chibobinizai	H	Whole plant	D	O	Dyspepsia, gastritis	4	0.020
HXZ-41004-10	*Stauntonia latifolia* (Wall.) R.Br	Lardizabalaceae	Bayuegua	Mainegeibomezai	WV	Fruit	D	O	Dyspepsia, edema, rheumatism	2	0.010
HXZ-41004-13	*Hypericum monogynum* L	Hypericaceae	Jinsitao	Cisidabosongzai	S	Root	D	O	Edema, pharyngitis, rheumatism	2	0.010
HXZ-41004-21	*Zanthoxylum bungeanum* Maxim	Rutaceae	Huajiao	Laigucuomezai	T	Fruit	D	O	Ascaridiasis, rheumatism, toothache	4	0.020
HXZ-41004-22	*Zanthoxylum acanthopodium* DC	Rutaceae	Cihuajiao	Cicuozai	T	Fruit	D	O	Rheumatism, stomachache	3	0.015
HXZ-41005-8	*Arisaema heterophyllum* Blume	Araceae	Tiannanxing	Haxingmezai	H	Tuber	D	O	Snake bite, stroke, traumatic injury	1	0.005
HXZ-41005-10	*Clerodendrum bungei* Steud	Lamiaceae	Choumudan	Anikoubozai	S	Leaf	D	O	Eczema, mastitis, rheumatism	1	0.005
HXZ-41005-12	*Pinus armandii* Franch	Pinaceae	Huashansong	Shumezai	T	Leaf	D	O	Cold, rheumatism	1	0.005
HXZ-41005-14	*Pyracantha fortuneana *(Maxim.) H.L.Li	Rosaceae	Huoji	Azimeguzai	S	Fruit	PU	O	Dyspepsia, metrorrhagia	1	0.005
HXZ-41005-22	*Dioscorea bulbifera* L	Dioscoreaceae	Huangdu	Zazongshanmezai	H	Tuber	D	O	Pharyngitis, whooping cough	2	0.010
HXZ-41005-34	*Daphne feddei* H.Lev	Thymelaeaceae	Dianruixiang	Puminangnangzai	S	Whole plant	I	O	Rheumatism, traumatic injury	2	0.010
HXZ-51006-9	*Cinnamomum aromaticum* Nees	Lauraceae	Guizhi	Bahuoxiangmizai	T	Bark	D	O	Dysmenorrhea, headache, lumbosacral pain	3	0.015
HXZ-41006-1	*Tetrastigma obtectum* Planch. ex Franch	Vitaceae	Yapateng	Mainaicibozai	HV	Whole plant	D	O	Rheumatism, traumatic injury	2	0.010
HXZ-41006-5	*Solanum nigrum* L	Solanaceae	Longkui	Laziwuzai	H	Whole plant	D	O	Cancer, dysentery, edema, pharyngitis, traumatic injury	1	0.005
HXZ-41006-11	*Dipsacus asper* Wall. ex DC	Caprifoliaceae	Xuduan	Huoshanpowoguozai	H	Root	I	O	Rheumatism, traumatic injury	6	0.031
HXZ-41006-12	*Cyrtomium caryotideum* C.Presl	Dryopteridaceae	Cichiguanzhong	Gongbengsizai	H	Root	D	EW	Snake bite, traumatic injury	2	0.010
HXZ-41006-19	*Fagopyrum acutatum* Mansf. ex K.Hammer	Polygonaceae	Jinqiaomai	Guozonggumezai	H	Whole plant	D	O	Diarrhea, hypertension, pharyngitis, pneumonia	5	0.026
HXZ-41006-22	*Girardinia diversifolia* (Link) Friis	Urticaceae	Daxiezicao	Anidoupuzai	H	Root	D	O	Fracture, migraine, rheumatism, stroke	3	0.015
HXZ-41006-23	*Cyathula officinalis* K.C.Kuan	Amaranthaceae	Chuanniuxi	Nisizai	H	Root	I	EA	Menostasis, rheumatism	5	0.026
HXZ-41006-24	*Alangium chinense* (Lour.) Harms	Cornaceae	Bajiaofeng	Abunitongzai	S	Root	I	EA	Rheumatism	2	0.010
HXZ-41006-26	*Geranium wilfordii* Maxim	Geraniaceae	Laoguancao	Wosizai	H	Whole plant	D	O	Mastitis, muscle and bone pain, stomachache	1	0.005
HXZ-70701-1	*Angelica sinensis* (Oliv.) Diels	Apiaceae	Danggui	Wonongzengmizai	H	Root	D	O	Dysmenorrhea, irregular menstruation, rheumatism	7	0.036
HXZ-41007-13	*Toddalia asiatica* (L.) Lam	Rutaceae	Feilongzhangxue	Nimenongtongzai	WV	Leaf	I	O	Lumbosacral pain, metrorrhagia, stomachache	4	0.020
HXZ-41018-2	*Cannabis sativa* L	Cannabaceae	Dama	Zimezai	H	Fruit	PW	O	Constipation, edema, epilepsy	2	0.010
HXZ-41116-14	*Coriaria nepalensis* Wall	Coriariaceae	Masang	Chibangzai	S	Leaf	PU	EA	Rheumatism	1	0.005
HXZ-70617-17	*Gastrodia elata* Bl	Orchidaceae	Tianma	Rizimetongzai	H	Tuber	PW	O	Migraine, rheumatism	3	0.015
HXZ-70702-6	*Rhododendron molle* (Blume) G.Don	Ericaceae	Naoyanghua	Cinaomilongshanzai	S	Root	D	O	Muscle and bone pain, rheumatism	1	0.005
HXZ-70624-4	*Orthosiphon wulfenioides* (Diels) Hand. -Mazz	Lamiaceae	Jijiaoshen	Rimunongzai	H	Root	PU	EA	Fracture, traumatic injury	1	0.005
HXZ-70521-14	*Spiranthes sinensis* (Pers.) Ames	Orchidaceae	Panlongshen	Agulonguzai	H	Whole plant	D	O	Burn and scald, nephritis, pharyngitis, snake bite	1	0.005
HXZ-41018-17	*Cynanchum otophyllum* C.K.Schneid	Apocynaceae	Qingyangshen	Cinaonongcizai	HV	Root	D	O	Lumbosacral pain, tinnitus	2	0.010
HXZ-41018-18	*Reynoutria japonica* Houtt	Polygonaceae	Huzhang	Luonizai	H	Root	D	EA	Burn and scald, rheumatism, traumatic injury	3	0.015
HXZ-41019-1	*Eleutherococcus senticosus* (Rupr. & Maxim.) Maxim	Araliaceae	Ciwujia	Ziwobuzai	S	Stem	PW	O	Rheumatism, traumatic injury	2	0.010
HXZ-50404-1	*Tinospora sagittata* var*. yunnanensis* (S.Y. Hu) H.S.Lo	Menispermaceae	Yunnanqingniudan	Nongcialaisongzai	HV	Root tuber	D	O	Mastitis, pharyngitis, rheumatism, stomachache	2	0.010
HXZ-41116-11	*Clematis chinensis* Osbeck	Ranunculaceae	Weilingxian	Hengnicibiezai	WV	Root	D	O	Lumbosacral pain, traumatic injury	3	0.015
HXZ-41019-24	*Prunella vulgaris* L	Lamiaceae	Xiakucao	Mehuosichizai	H	Whole plant	D	O	Cancer, hypertension, mastitis, pharyngitis	3	0.015
HXZ-51018-20	*Curcuma longa* L	Zingiberaceae	Jianghuang	Chiboshanzai	H	Rhizome	PW	O	Rheumatism, traumatic injury	4	0.020
HXZ-51114-7	*Gynura segetum* (Lour.) Merr	Asteraceae	Jusanqi	Ninongnongcizai	H	Whole plant	PU	EA	Lumbosacral pain, traumatic injury	2	0.010
HXZ-41102-16	*Laggera pterodonta* (DC.) Benth	Asteraceae	Choulingdan	Chikaozai	H	Whole plant	PU	EA	Parotitis, scabies	4	0.020
HXZ-41115-4	*Oxalis corniculata* L	Oxalidaceae	Cujiangcao	Simezimezai	H	Whole plant	PU	EA	Burn and scald, eczema, scabies, snake bite	1	0.005
HXZ-41115-12	*Litsea rubescens* Lecomte	Lauraceae	Hongyemujiangzi	Cuogumezai	S	Root	D	O	Cold, headache, rheumatism	2	0.010
HXZ-41115-17	*Heracleum scabridum* Franch	Apiaceae	Caoduhuo	Xiangxiangtongmezai	H	Root	D	O	Rheumatism, stomachache	1	0.005
HXZ-41116-4	*Hemiphragma heterophyllum* Wall	Plantaginaceae	Biandaxiuqiu	Alainibaizai	H	Whole plant	D	O	Menostasis, pharyngitis, rheumatism	1	0.005
HXZ-70826-3	*Centella asiatica* (L.) Urban	Apiaceae	Jixuecao	Mengkongwuzai	H	Whole plant	D	O	Hepatitis, traumatic injury	3	0.015
HXZ-50405-19	*Psammosilene tunicoides* W.C.Wu & C.Y.Wu	Caryophyllaceae	Jintiesuo	Anijingsongzai	H	Root	D	O	Stomachache, traumatic injury	4	0.020
HXZ-50502-5	*Heptapleurum arboricola* Hayata	Araliaceae	Qiyelian	Shitognpiezai	S	Leaf	PU	EA	Fracture, traumatic injury	4	0.020
HXZ-51018-13	*Panax notoginseng* (Burkill) F.H.Chen	Araliaceae	Sanqi	Senbinimezai	H	Root	PW	O	Dysmenorrhea, irregular menstruation, traumatic injury	6	0.031
HXZ-51115-4	*Aconitum vilmorinianum* Kom	Ranunculaceae	Huangcaowu	Ciduzai	H	Root tuber	I	EA	Rheumatism, traumatic injury	3	0.015
HXZ-70617-20	*Acorus calamus* var*. angustatus* Besser	Acoraceae	Shichangpu	Luomebinengsizai	H	Rhizome	D	O	Dyspepsia, gastritis, lumbosacral pain	2	0.010
HXZ-70319-2	*Chaenomeles sinensis* (Thouin) Koehne	Rosaceae	Mugua	Siapucimezai	T	Fruit	D	O	Dyspepsia, edema, lumbosacral pain, rheumatism	4	0.020
HXZ-61003-13	*Piper betle* L	Piperaceae	Louye	Heicuonianmezai	WV	Leaf	D	O	Asthma, cold, dyspepsia, eczema	1	0.005
HXZ-61113-18	*Gardenia jasminoides *J.Ellis	Rubiaceae	Zhizi	Shanlaisangeizai	S	Fruit	D	O	Burn and scald, cold, nephritis, pharyngitis	1	0.005
HXZ-70617-11	*Scleromitrion diffusum* (Willd.) R.J.Wang	Rubiaceae	Baihuasheshecao	Hanmetongsizai	H	Whole plant	D	O	Dysentery, hepatitis, pharyngitis	1	0.005
HXZ-41116-6	*Lycopodium japonicum* Thunb	Lycopodiaceae	Shenjincao	Laibumianhaozai	H	Whole plant	D	O	Rheumatism, traumatic injury	10	0.051
HXZ-41116-7	*Phryma leptostachya* L	Phrymaceae	Tougucao	Nainzhazai	H	Whole plant	D	EW	Eczema, scabies	6	0.031
HXZ-80815-24	*Polygonum paleaceum* Wall	Polygonaceae	Caoxuejie	Daikabunianyucizai	H	Rhizome	D	O	Dyspepsia, metrorrhagia, traumatic injury	4	0.020
HXZ-41019-7	*Cinnamomum camphora* (L.) J.Presl	Lauraceae	Zhang	Motongnianmezai	T	Branch	D	EW	Rheumatism, scabies	1	0.005
HXZ-41102-24	*Amomum tsaoko* Crevost et Lemarie	Zingiberaceae	Caoguo	Luhaobinengzai	H	Fruit	D	O	Dyspepsia	1	0.005
HXZ-50503-14	*Hovenia acerba* Lindl	Rhamnaceae	Zhiju	Guluguloumezai	T	Seed	I	O	Rheumatism	1	0.005
HXZ-71003-31	*Sarcandra glabra* (Thunb.) Nakai	Chloranthaceae	Caoshanhu	Sichongnianzai	S	Whole plant	D	O	Cold, pneumonia, rheumatism	2	0.010
HXZ-51122-7	*Galium elegans* Wall	Rubiaceae	Xiaohongshen	Nibaiadaizai	H	Root	D	O	Irregular menstruation, rheumatism	4	0.020
HXZ-60911-2	*Plumbago zeylanica* L	Plumbaginaceae	Baihuadan	Tongmilongzai	S	Whole plant	D	EW	Snake bite, traumatic injury	2	0.010
HXZ-41116-10	*Valeriana jatamansi* Jones	Caprifoliaceae	Zhizhuxiang	Gongbunizai	H	Root	D	O	Dyspepsia, irregular menstruation	4	0.020
HXZ-50405-11	*Prunus sibirica* L	Rosaceae	Xingren	Sangannizai	T	Seed	PW	O	Constipation, pharyngitis	1	0.005
HXZ-41005-29	*Dioscorea polystachya* Turcz	Dioscoreaceae	Shanyao	Laiguagazai	HV	Rhizome	D	O	Dyspepsia, nephritis	2	0.010
HXZ-41115-22	*Corydalis taliensis* Franch	Papaveraceae	Jingouruyicao	Yuzhongsinenzai	H	Whole plant	D	O	Dysentery, hepatitis, rheumatism, toothache	1	0.005
HXZ-51006-11	*Spatholobus suberectus* Dunn	Fabaceae	Jixueteng	Wocaisinangzai	WV	Stem	D	O	Dysmenorrhea, headache, rheumatism	1	0.005
HXZ-60911-1	*Machilus yunnanensis* Lec	Lauraceae	Gouzhaozhangpi	Wodongtongzai	T	Bark	D	O	Diarrhea	1	0.005
HXZ-51001-5	*Zingiber officinale* Rosc	Zingiberaceae	Ganjiang	Chibozai	H	Rhizome	D	O	Diarrhea, rheumatism	14	0.071
HXZ-50328-26	*Sida szechuensis* Matsuda	Malvaceae	Badusan	Geipengnongcizai	S	Leaf	D	O	Diarrhea, mastitis, menostasis	2	0.010
HXZ-50405-10	*Stephania epigaea* H.S.Lo	Menispermaceae	Diburong	Anibadaizai	HV	Root tuber	PU	EA	Snake bite	1	0.005
HXZ-51018-12	*Coix lacryma-jobi* L	Poaceae	Yiyi	Dangheimezai	H	Seed	D	O	Dyspepsia	4	0.020
HXZ-50524-10	*Urtica atrichocaulis* (Hand.-Mazz.) C. J. Chen	Urticaceae	Xiaoguoqianma	Doupuazeizai	H	Whole plant	D	O	Lumbosacral pain	1	0.005
HXZ-61001-2	*Sambucus javanica* Reinw. ex Blume	Viburnaceae	Luying	Zimeniantongzai	H	Leaf	D	O	Edema, lumbosacral pain	1	0.005
HXZ-41018-11	*Akebia quinata* (Thunb.) Decne	Lardizabalaceae	Mutong	Musaigeizai	WV	Stem	D	O	Dysmenorrhea, edema, menostasis, pharyngitis	1	0.005
HXZ-50419-11	*Pueraria lobata* (Willd.) Ohwi	Fabaceae	Gegen	Cinaigaiguozai	HV	Root	PW	O	Headache, hypertension, tinnitus	4	0.020
HXZ-41102-18	*Morus alba* L	Moraceae	Sangshu	Buchegutongzai	T	Leaf	D	O	Cold, edema, headache, pharyngitis	2	0.010
HXZ-41130-8	*Paederia foetida* L	Rubiaceae	Jishiteng	Yankoubinizai	HV	Whole plant	D	O	Dyspepsia, fracture, hepatitis	3	0.015
HXZ-41006-4	*Helwingia himalaica* Hook.f. & Thomson ex C.B.Clarke	Helwingiaceae	Yeshangguo	Babamezai	S	Root	I	O	Irregular menstruation, stomachache, traumatic injury	2	0.010
HXZ-41005-3	*Lysimachia paridiformis *Franch	Primulaceae	Sikuaiwa	Lipianzai	H	Whole plant	D	O	Rheumatism, snake bite, traumatic injury	4	0.020
HXZ-41005-4	*Paris polyphylla* var*. yunnanensis* (Franch.) Hand.-Mzt	Melanthiaceae	Zhonglou	Ameichebuzai	H	Rhizome	PW	O	Pharyngitis, snake bite, traumatic injury	5	0.026
HXZ-41005-36	*Eriocapitella rivularis* (Buch.-Ham. ex DC.) Christenh. & Byng	Ranunculaceae	Huzhangcao	Woguowonizai	H	Root	I	O	Hepatitis, pharyngitis, toothache, traumatic injury	3	0.015
HXZ-41006-10	*Gnaphalium affine* D. Don	Asteraceae	Shuqucao	Ageizonglongsongzai	H	Whole plant	D	O	Bronchitis, rheumatism	2	0.010
HXZ-41116-1	*Leycesteria formosa* Wall	Caprifoliaceae	Guichuixiao	Niannuoazitezai	S	Whole plant	D	O	Asthma, edema, irregular menstruation, rheumatism	1	0.005
HXZ-41116-9	*Erigeron breviscapus* (Vant.) Hand. -Mazz	Asteraceae	Dengzhanxixin	Sizaiwuzai	H	Whole plant	D	O	Headache, rheumatism, toothache	1	0.005
HXZ-41116-13	*Aristolochia yunnanensis* Franch	Aristolochiaceae	Xiaonanmuxiang	Nangtongzai	WV	Root	I	O	Dyspepsia, rheumatism, traumatic injury	2	0.010
HXZ-70701-4	*Typhonium giganteum* Engl	Araceae	Dujiaolian	Abutongnizai	H	Whole plant	PU	EA	Snake bite, traumatic injury	2	0.010
HXZ-41004-14	*Pinellia ternata* (Thunb.) Makino	Araceae	Banxia	Haliangusongzai	H	Tuber	D	O	Dyspepsia	4	0.020
HXZ-50405-4	*Wisteria brachybotrys* Siebold & Zucc	Fabaceae	Dafahan	Wozhangdoumezai	WV	Root	PW	O	Cold, rheumatism	1	0.005
HXZ-50405-12	*Berchemia floribunda* (Wall.) Brongn	Rhamnaceae	Huangshanteng	Mengzizai	S	Root	PU	EA	Fracture	1	0.005
HXZ-50405-23	*Cynoglossum amabile* Stapf & J.R.Drumm	Boraginaceae	Daotihu	Nongkegazai	H	Whole plant	PU	EA	Fracture	1	0.005
HXZ-50406-5	*Houttuynia cordata* Thunb	Saururaceae	Yuxingcao	Awobinengzai	H	Leaf	D	O	Edema, malaria, pneumonia	4	0.020
HXZ-50524-6	*Artemisia argyi* Levl.et Vant	Asteraceae	Aihao	Wosuoakongzai	H	Leaf	D	O	Dysmenorrhea, irregular menstruation, scabies	2	0.010
HXZ-51003-4	*Xanthium sibiricum *Patrin ex Widder	Asteraceae	Cangerzi	Wocenzisanzai	H	Fruit	D	O	Parotitis, rhinitis, toothache	1	0.005
HXZ-51006-4	*Strychnos nux-vomica* L	Loganiaceae	Maqianzi	Monaomezai	T	Seed	PW	O	Rheumatism, traumatic injury	1	0.005
HXZ-51025-2	*Anisodus acutangulus* C. Y. Wu et C. Chen	Solanaceae	Sanfensan	Senfensenfenzai	H	Leaf	D	O	Lumbosacral pain, rheumatism, stomachache	1	0.005
HXZ-41115-10	*Craibiodendron yunnanense* W. W. Sm	Ericaceae	Jinyezi	Shanpiantongzai	T	Leaf	PW	O	Cold, rheumatism, traumatic injury	1	0.005
HXZ-61106-29	*Orthosiphon aristatus* var*. aristatus*	Lamiaceae	Maoxucao	Meinainiancengzai	H	Leaf	D	O	Nephritis, rheumatism, stone	2	0.010
HXZ-50508-1	*Liquidambar formosana *Hance	Altingiaceae	Lulutong	Zhongsongzhongmangzai	T	Fruit	D	O	Edema, rheumatism	1	0.005
HXZ-60911-12	*Ligusticum striatum* DC	Apiaceae	Chuanxiong	Yansonganengzai	H	Rhizome	D	O	Dysmenorrhea, headache, irregular menstruation, menostasis, rheumatism	5	0.026
HXZ-61119-3	*Cymbopogon citratus* Stapf	Poaceae	Xiangmao	Mainaibinengzai	H	Whole plant	D	O	Diarrhea, headache, irregular menstruation, stomachache, traumatic injury	1	0.005
HXZ-61106-10	*Parochetus communis* Buch.-Ham. ex D.Don	Fabaceae	Jinquehua	Azhongshanmizai	H	Whole plant	PU	EA	Traumatic injury	2	0.010
HXZ-70625-4	*Aconitum racemulosum* Franch	Ranunculaceae	Xueshangyizhihao	Ciduzai	H	Root tuber	I	EA	Rheumatism, traumatic injury	4	0.020
HXZ-80815-5	*Aconitum carmichaelii* Debeaux	Ranunculaceae	Wutou	Acidu	H	Root tuber	I	EA	Rheumatism, stroke, traumatic injury	7	0.036
HXZ-41007-5	*Delphinium yunnanense* Franch	Ranunculaceae	Yunnancuiquehua	Cidunanazai	H	Root tuber	I	EA	Traumatic injury	2	0.010
HXZ-51107-4	*Tinospora sinensis* (Lour.) Merr	Menispermaceae	Zhonghuaqingniudan	Lamesizai	HV	Root tuber	I	EA	Traumatic injury	1	0.005
HXZ-41025-4	*Smilax mairei* H.Lev	Smilacaceae	Hongbixie	Ziaduomegulianzai	S	Rhizome	D	O	Edema, gastritis, nephritis, rheumatism	1	0.005
HXZ-51001-4	*Flemingia prostrata* Roxb.Junior ex Roxb	Fabaceae	Qianjinba	Qianduozizai	S	Root	D	O	Pharyngitis, rheumatism, traumatic injury	3	0.015
HXZ-41003-1	*Viburnum foetidum* Wall	Viburnaceae	Laomijiushu	Gongbusizhazai	S	Leaf	PU	EA	Fracture, traumatic injury	1	0.005
HXZ-50419-7	*Solanum donianum* Walp	Solanaceae	Yeqieshu	Yangumezai	H	Root	PU	EA	Fracture	1	0.005
HXZ-61113-13	*Schisandra chinensis* (Turcz.) Baill	Schisandraceae	Wuweizi	Sangawominzai	WV	Fruit	D	O	Diarrhea	1	0.005
HXZ-41001-02	*Carpesium abrotanoides* L	Asteraceae	Tianmingjing	Zhaosongsizai	H	Whole plant	PU	EA	Snake bite	2	0.010
HXZ-41001-06	*Lonicera maackii* (Rupr.) Maxim	Caprifoliaceae	Jinyinrendong	Wogongzai	S	Flower	D	O	Cold, eczema, pharyngitis	3	0.015
HXZ-410-01-07	*Pyrus pashia* Buch. -Ham. ex D. Don	Rosaceae	Chuanli	Sanlimianliansongzai	T	Fruit	PU	O	Diarrhea, dyspepsia	1	0.005
HXZ-410-01-08	*Eupatorium heterophyllum* DC	Asteraceae	Hongshengma	Woguonongcizai	H	Whole plant	D	O	Edema, irregular menstruation	3	0.015
HXZ-410-02-07	*Swertia mileensis* T.N.Ho & W.L.Shih	Gentianaceae	Mengzizhangyacai	Sikaozai	H	Whole plant	D	O	Dyspepsia, toothache	1	0.005
HXZ-410-02-15	*Eremochloa ciliaris* (L.) Merr	Poaceae	Wugongcao	Heihuomanzhizai	H	Whole plant	D	O	Cold, dysentery, rheumatism	1	0.005
HXZ-410-02-21	*Chrysojasminum subhumile* (W.W.Sm.) Banfi & Galasso	Oleaceae	Diansuxin	Nizixingmezai	S	Leaf	D	O	Cold, headache, rheumatism	1	0.005
HXZ-410-02-30	*Elsholtzia rugulosa* Hemsl	Lamiaceae	Yebazi	Asaizai	H	Whole plant	D	O	Cold, dysentery, dyspepsia	4	0.020
HXZ-410-02-31	*Sarcococca ruscifolia* Stapf	Buxaceae	Yeshanhua	Wobonongqizai	S	Fruit	D	O	Palpitation	1	0.005
HXZ-410-02-33	*Persicaria capitata* (Buch.-Ham. ex D.Don) H.Gross	Polygonaceae	Touhualiao	Woguomilongduozai	H	Whole plant	PU	EA	Traumatic injury	1	0.005
HXZ-410-03-07	*Pyrus pyrifolia* (Burm. f.) Nakai	Rosaceae	Shali	Sanlimianlianerzai	T	Fruit	PU	O	Cough	1	0.005
HXZ-410-03-08	*Mirabilis jalapa* L	Nyctaginaceae	Zimoli	Lazishanguzai	H	Root tuber	D	O	Irregular menstruation, traumatic injury	2	0.010
HXZ-410-03-12	*Urtica fissa* E.Pritz. ex Diels	Urticaceae	Qianma	Doupuzai	H	Whole plant	D	O	Constipation, dyspepsia, traumatic injury	1	0.005
HXZ-410-04-05	*Colocasia esculenta* (L). Schott	Araceae	Yu	Abunibaizai	H	Tuber	D	O	Burn and scald, mastitis	1	0.005
HXZ-410-04-09	*Justicia procumbens* L	Acanthaceae	Juechuang	Citusizai	H	Whole plant	D	O	Cold, hepatitis, pharyngitis	1	0.005
HXZ-410-04-16	*Siegsbeckia orientalis* L	Asteraceae	Xixiancao	Nijiezai	H	Leaf	D	O	Rheumatism	1	0.005
HXZ-410-04-18	*Thelypteris gongylodes* (Schkuhr) Small	Thelypteridaceae	Maojue	Abiwujiezai	H	Whole plant	D	O	Rheumatism	1	0.005
HXZ-410-04-23	*Sophora davidii* (Franch.) Skeels	Fabaceae	Baicihua	Longzizizai	S	Flower	D	O	Dysentery, edema	2	0.010
HXZ-410-04-27	*Piloselloides hirsuta* (Forssk.) C.Jeffrey ex Cufod	Asteraceae	Maodadingcao	Yanpubanlaisongzai	H	Whole plant	D	O	Asthma, cold, edema	1	0.005
HXZ-410-05-19	*Biancaea decapetala* (Roth) O.Deg	Fabaceae	Yunshi	Luozhizai	WV	Root	D	O	Cold, dysentery, lumbosacral pain, toothache	2	0.010
HXZ-410-05-25	*Musella lasiocarpa* (Franch.) H.W.Li	Musaceae	Diyongjinlian	Shanpianwuzai	H	Flower	D	O	Metrorrhagia	1	0.005
HXZ-410-05-28	*Sapindus delavayi* (Franch.) Radlk	Sapindaceae	Chuandianwuhuanzi	Mianlimezai	T	Fruit	PU	O	Cough	1	0.005
HXZ-410-05-31	*Polygonatum sibiricum* F.Delaroche	Asparagaceae	Huangjing	Bilangongdaizai	H	Rhizome	D	O	Cough, tinnitus	2	0.010
HXZ-410-05-33	*Viola yunnanfuensis* W.Becker	Violaceae	Ziluolan	Nibaizongduozai	H	Whole plant	PU	EA	Scabies	1	0.005
HXZ-410-05-35	*Polygala arillata* Buch.-Ham. ex D. Don	Polygalaceae	Hebaoshanguihua	Yansonggongdengzai	T	Root	D	O	Hepatitis, nephritis, pneumonia, traumatic injury	1	0.005
HXZ-410-05-41	*Rhus chinensis* Mill	Anacardiaceae	Yanfumu	Sanmodanlazai	T	Flower	PU	EA	Scabies	1	0.005
HXZ-410-06-04	*Helwingia himalaica* Hook. f. et Thoms. ex C. B. Clarke	Helwingiaceae	Xiyuqingjiaye	Babamezai	S	Leaf	D	O	Cold, dysentery, fracture, rheumatism, snake bite, stomachache	1	0.005
HXZ-410-06-06	*Agrimonia pilosa* var*. nepalensis* (D.Don) Nakai	Rosaceae	Huanglongwei	Wosailongsizai	H	Whole plant	D	O	Dysentery, traumatic injury	2	0.010
HXZ-410-06-07	*Cirsium japonicum* DC	Asteraceae	Ji	Shanpaizizai	H	Whole plant	D	O	Metrorrhagia, parotitis, traumatic injury	1	0.005
HXZ-410-06-27	*Eriocapitella vitifolia* (Buch.-Ham. ex DC.) Nakai	Ranunculaceae	Yemianhua	Gabiyansizai	H	Root	D	O	Ascaridiasis, traumatic injury	1	0.005
HXZ-410-07-16	*Oreocnide frutescens* (Thunb.) Miq	Urticaceae	Shuima	Chibangzai	S	Whole plant	PU	EA	Rheumatism, scabies	2	0.010
HXZ-41007-3	*Cornus kousa* subsp. chinensis (Osborn) Q.Y.Xiang	Cornaceae	Sizhaohua	Sanzizai	T	Fruit	D	O	Ascaridiasis	1	0.005
HXZ-410-18-03	*Capsella bursa-pastoris* Medik	Brassicaceae	Qi	Ziwuzai	H	Whole plant	D	O	Dyspepsia, headache	2	0.010
HXZ-410-18-06	*Thesium refractum* C.A.Mey	Santalaceae	Jizhebairuicao	Geisaisizai	H	Whole plant	D	O	Pharyngitis, pneumonia	1	0.005
HXZ-410-18-08	*Stellaria yunnanensis* Franch	Caryophyllaceae	Qianzhenwanxiancao	Qianduowasizai	H	Root	D	O	Fracture, mastitis, palpitation	2	0.010
HXZ-410-18-12	*Merremia martini* (H.Lev.) Staples & Simoes	Convolvulaceae	Shantugua	Laiguanaizai	HV	Root tuber	D	O	Cough, hepatitis	1	0.005
HXZ-410-19-05	*Taraxacum mongolicum* Hand.-Mazz	Asteraceae	Pugongying	Yanshuowutongzai	H	Whole plant	D	O	Cold, hepatitis, mastitis, pharyngitis	5	0.026
HXZ-410-19-09	*Crotalaria albida* B. Heyne ex Roth	Fabaceae	Xianglingdou	Mudengzhalazai	H	Whole plant	PU	EA	Traumatic injury	1	0.005
HXZ-410-19-18	*Gonostegia hirta* Miq	Urticaceae	Nuomituan	Zongniananangnangzai	H	Whole plant	D	O	Dysmenorrhea, dyspepsia, edema	1	0.005
HXZ-410-19-19	*Scutellaria orthocalyx* Hand.-Mazz	Lamiaceae	Xiaohuangqin	Cimeshanzai	H	Whole plant	D	O	Pharyngitis, scabies	2	0.010
HXZ-410-19-20	*Myrica nana* A.Chev	Myricaceae	Yunnanyangmei	Sangusongzai	S	Bark	PW	O	Diarrhea, traumatic injury	1	0.005
HXZ-410-19-25	*Silene baccifera* Roth	Caryophyllaceae	Goujinman	Niantongnongzai	H	Root	PU	EA	Rheumatism, traumatic injury	1	0.005
HXZ-410-25-02	*Pistacia weinmannifolia* J.Poiss. ex Franch	Anacardiaceae	Qingxiangmu	Luozongsizai	T	Leaf	D	O	Pharyngitis	3	0.015
HXZ-410-2505	*Platycarya strobilacea* Siebold & Zucc	Juglandaceae	Huaxiangshu	Woluoduosizai	T	Leaf	PU	EA	Scabies	1	0.005
HXZ-410-25-08	*Ficus ti-koua* Bureau	Moraceae	Diguo	Cisanpianlianzai	WV	Whole plant	D	O	Edema, pharyngitis, rheumatism	1	0.005
HXZ-410-25-09	*Mesosphaerum pectinatum* Kuntze	Lamiaceae	Zisu	Nihengmezai	H	Leaf	D	O	Cold, cough	4	0.020
HXZ-410-25-14	*Ricinus communis* L	Euphorbiaceae	Bima	Mazizai	H	Root	D	O	Epilepsy, rheumatism, tetanus	1	0.005
HXZ-410-25-16	*Osyris lanceolata* Hochst. & Steud	Santalaceae	Shazhen	Songpouxiangmizai	S	Root	PW	EA	Traumatic injury	1	0.005
HXZ-411-02-03	*Lagenaria siceraria* (Molina) Standl	Cucurbitaceae	Hulu	Apumuguoluozai	HV	Fruit	D	O	Edema	1	0.005
HXZ-411-02-04	*Lablab purpureus* (L.) Sweet	Fabaceae	Biandou	Anujiezai	HV	Seed	D	O	Diarrhea	2	0.010
HXZ-411-02-07	*Buddleja officinalis* Maxim	Scrophulariaceae	Mimenghua	Woguozai	S	Flower	D	O	Eye disease	2	0.010
HXZ-411-02-09	*Rumex hastatus* D. Don	Polygonaceae	Jiyesuanmo	Zimeshanzai	S	Whole plant	D	O	Cold, cough, edema	1	0.005
HXZ-411-02-10	*Melastoma malabathricum* L	Melastomataceae	Yemudan	Mukangtabaozai	S	Root	D	O	Dysentery, dyspepsia, hepatitis	1	0.005
HXZ-411-02-17	*Phytolacca acinosa* Roxb	Phytolaccaceae	Shanglu	Cimimezai	H	Root	PU	EA	Dermatophytosis, edema	1	0.005
HXZ-411-02-19	*Lobelia nummularia* Lam	Campanulaceae	Tongchuiyudaicao	Zilusizai	H	Fruit	D	O	Rheumatism, traumatic injury	1	0.005
HXZ-411-02-29	*Jasminum nudiflorum* Lindl	Oleaceae	Yingchunhua	Miluokaozhizai	S	Leaf	D	O	Cold, traumatic injury	1	0.005
HXZ-411-14-05	*Datura stramonium* L	Solanaceae	Mantuoluo	Cishanmiluozai	H	Leaf	D	EW	Dermatophytosis, rheumatism	2	0.010
HXZ-411-14-08	*Euphorbia lathyris* L	Euphorbiaceae	Xusuizi	Wobenggeitongzai	H	Leaf	PU	EA	Edema, snake bite	1	0.005
HXZ-411-15-06	*Stellaria aquatica* Scop	Caryophyllaceae	Echangcai	Ouniwuzai	H	Whole plant	D	O	Rheumatism, tuberculosis	1	0.005
HXZ-411-15-08	*Lobelia seguinii* H.Lev. & Vaniot	Campanulaceae	Xinanshangengcai	Nonggongpocibengzai	S	Root	PU	EA	Rheumatism, traumatic injury	1	0.005
HXZ-411-15-16	*Ainsliaea yunnanensis* Franch	Asteraceae	Yunnantuerfeng	Aluosongsizai	H	Whole plant	PU	EA	Fracture, rheumatism	1	0.005
HXZ-411-15-18	*Hemsleya sphaerocarpa* Kuang & A.M.Lu	Cucurbitaceae	Shelian	Hameliankaozai	HV	Rhizome	PU	EA	Snake bite, traumatic injury	1	0.005
HXZ-411-15-23	*Rodgersia sambucifolia* Hemsl	Saxifragaceae	Xinanguidengqing	Laibuchibotongzai	H	Rhizome	I	O	Fracture, rheumatism	2	0.010
HXZ-411-16-16	*Crepis phoenix* Dunn	Asteraceae	Wanzhangshen	Qiandongnianzai	H	Root	I	O	Bronchitis, hepatitis, lumbosacral pain, pneumonia	1	0.005
HXZ-411-16-17	*Sambucus williamsii* Hance	Viburnaceae	Jiegumu	Eguozezai	S	Whole plant	D	O	Nephritis, rheumatism, traumatic injury	3	0.015
HXZ-411-30-09	*Dichondra micrantha* Urb	Convolvulaceae	Matijin	Maokongnangwuzai	H	Whole plant	D	O	Dysentery, hepatitis, nephritis	1	0.005
HXZ-503--21-08	*Hedera sinensis* (Tobler) Hand.-Mazz	Araliaceae	Changchunteng	Hanxingmenazhongzai	WV	Leaf	PW	O	Hepatitis, irregular menstruation, pharyngitis	1	0.005
HXZ-503-21-12	*Eriobotrya japonica* (Thunb.) Lindl	Rosaceae	Pipa	Anipiansetongzai	T	Leaf	D	O	Cough	3	0.015
HXZ-503-21-16	*Maclura tricuspidata* Carriere	Moraceae	Tuoshu	Mocuodoumezai	T	Leaf	D	O	Eczema, parotitis, tuberculosis	1	0.005
HXZ-503-21-17	*Diospyros yunnanensis* Rehder & E.H.Wilson	Ebenaceae	Yunnanshi	Laigusanbaozai	T	Fruit	PU	O	Cough	1	0.005
HXZ-503-28-01	*Trachycarpus fortunei* (Hook.) H.Wendl	Arecaceae	Zonglü	Sitongzai	T	Flower	D	O	Dysentery	1	0.005
HXZ-503-28-03	*Plantago asiatica* L	Plantaginaceae	Cheqian	Abengwujiezai	H	Whole plant	D	O	Cough, edema, eye disease	5	0.026
HXZ-503-28-04	*Magnolia officinalis* Rehder & E.H.Wilson	Magnoliaceae	Houpu	Dabaotongzai	T	Bark	D	O	Constipation	1	0.005
HXZ-503-28-13	*Periploca calophylla* (Wight) Falc	Apocynaceae	Qingsheteng	Hanmenianchizai	S	Stem	I	O	Lumbosacral pain, snake bite	2	0.010
HXZ-503-28-19	*Gynostemma pentaphyllum* (Thunb.) Makino	Cucurbitaceae	Jiaogulan	Nongpianwopianzai	HV	Whole plant	D	O	Bronchitis, gastritis, hepatitis	2	0.010
HXZ-503-28-23	*Pholidota chinensis* Lindl	Orchidaceae	Shixiantao	Ludougumezai	H	Whole plant	D	O	Cough, pharyngitis, traumatic injury	1	0.005
HXZ-504-04-08	*Mahonia bealei* (Fortune) Carriere	Berberidaceae	Kuoyeshidagonglao	Zimeshanzai	T	Leaf	D	O	Diarrhea, eye disease, hepatitis, tinnitus	5	0.026
HXZ-504-0411-	*Senecio scandens* (L.) Buch.-Ham	Asteraceae	Luojingqianliguang	Minjienigeitongzai	H	Whole plant	D	O	Dyspepsia, fracture, irregular menstruation	2	0.010
HXZ-504-04-12	*Selaginella pulvinata* (Hook. et Grev.) Maxim	Selaginellaceae	Dianzhuangjuanbai	Laipisainongzai	H	Whole plant	D	O	Traumatic injury	1	0.005
HXZ-504-0503	*Prunus persica* (L.) Batsch	Rosaceae	Tao	Wosuosanwuzai	T	Leaf	D	EW	Eczema, rheumatism	1	0.005
HXZ-504-05-20	*Platycladus orientalis* (L.) Franco	Cupressaceae	Cebai	Shujietongzai	T	Leaf	D	EW	Burn and scald, rheumatism, scabies	2	0.010
HXZ-504-05-21	*Ficus carica* Linn	Moraceae	Wuhuaguo	Amimezai	T	Fruit	D	O	Constipation, dyspepsia, pharyngitis	1	0.005
HXZ-504-05-24	*Verbena officinalis* L	Verbenaceae	Mabiancao	Mobiansizai	H	Whole plant	D	O	Edema, malaria, pharyngitis	3	0.015
HXZ-504-06-16	*Bupleurum marginatum *Wall. ex DC	Apiaceae	Zhuyechaihu	Sizaisibizai	H	Whole plant	D	O	Cold, irregular menstruation	2	0.010
HXZ-504-06-17	*Amorphophallus konjac* K. Koch	Araceae	Moyu	Moyuzai	H	Tuber	PU	EA	Burn and scald, snake bite, traumatic injury	1	0.005
HXZ-504-18-01	*Xylanche himalaica* (Hook.f. & Thomson) Beck	Orobanchaceae	Dingzuocao	Akongtongtongzai	H	Tuber	PW	O	Cough, dyspepsia	1	0.005
HXZ-504-18-16	*Ulmus pumila* L	Ulmaceae	Yushu	Arusizai	T	Bark	I	EA	Fracture	1	0.005
HXZ-504-18-17	*Rhus chinensis* Mill	Anacardiaceae	Qingfuyang	Sanmodanlazai	T	Root	D	EW	Scabies, traumatic injury	1	0.005
HXZ-504-19-02	*Polygala sibirica* L	Polygalaceae	Xiboliyayuanzhi	Midousizai	H	Root	D	O	Cough, edema	1	0.005
HXZ-504-19-03	*Salvia yunnanensis* C. H. Wright	Lamiaceae	Yunnanshuweicao	Binglangsizai	H	Root	D	O	Dysmenorrhea	2	0.010
HXZ-504-19-07	*Solanum erianthum* D.Don	Solanaceae	Jiayanyeshu	Yangumezai	S	Leaf	D	O	Eczema, edema, toothache	1	0.005
HXZ-504-19-12	*Punica granatum* L	Lythraceae	Shiliu	Sanbu	T	Flower	D	O	Irregular menstruation, toothache	1	0.005
HXZ-504-19-14	*Sophora flavescens* Aiton	Fabaceae	Kushen	Mengzhongkaozhizai	S	Root	D	EW	Bronchitis, eczema	3	0.015
HXZ-504-19-20	*Rhaphidophora peepla* Schott	Araceae	Pashulong	Hanmenibizai	WV	Whole plant	D	O	Bronchitis, fracture, whooping cough	1	0.005
HXZ-505-02-14	*Stephania delavayi* Diels	Menispermaceae	Xianhuaqianjinteng	Yumeawenwenzai	HV	Root	D	O	Cold, pharyngitis	1	0.005
HXZ-505-02-15	*Talinum paniculatum* (Jacq.) Gaertn	Talinaceae	Turenshen	Dengpuzaizai	H	Root	D	O	Cough, diarrhea, irregular menstruation	1	0.005
HXZ-50503-10	*Leonurus japonicus* Houtt	Lamiaceae	Yimucao	Ametongsongzai	H	Whole plant	D	O	Irregular menstruation, traumatic injury	6	0.031
HXZ-505-08-04	*Albizia kalkora* (Roxb.) Prain	Fabaceae	Shanhuai	Cinicengzai	T	Bark	D	EW	Traumatic injury	1	0.005
HXZ-505-09-06	*Liriope spicata* (Thunb.) Lour	Asparagaceae	Shanmaidong	Siyanmesongzai	H	Root tuber	D	O	Constipation, cough	1	0.005
HXZ-505-10-05	*Phragmites australis* (Cav.) Steud	Poaceae	Luwei	Rimesisongzai	H	Root	D	O	Constipation	1	0.005
HXZ-505-10-08	*Imperata cylindrica* (L.) P.Beauv	Poaceae	Baimao	Luoshizai	H	Root	D	O	Cough, edema	4	0.020
HXZ-505-23-13	*Cuscuta australis* R.Br	Convolvulaceae	Tusizi	Ameabuzai	H	Seed	D	O	Lumbosacral pain, tinnitus	3	0.015
HXZ-50524-01	*Vincetoxicum yunnanense* (Schltr.) Meve & Liede	Apocynaceae	Yunnanwaerteng	Nimeanengbinazai	S	Root	D	O	Hepatitis, malaria, rheumatism	1	0.005
HXZ-50530-05	*Ophiopogon japonicus* (Thunb.) Ker Gawl	Asparagaceae	Maidong	Siyanmemezai	H	Root tuber	D	O	Constipation, cough, pharyngitis	2	0.010
HXZ-505-30-07	*Euonymus grandiflorus* Wall	Celastraceae	Dahuaweimao	Shantongyanmanzai	T	Bark	D	O	Dysentery, rheumatism	1	0.005
HXZ-505-30-08	*Eucommia ulmoides *Oliver	Eucommiaceae	Duzhong	Yanmantongmezai	T	Bark	D	O	Hypertension, lumbosacral pain	2	0.010
HXZ-505-30-14	*Juncus effusus* L	Juncaceae	Dengxincao	Mozhasizai	H	Stem	D	O	Pharyngitis	1	0.005
HXZ-505-31-03	*Boenninghausenia albiflora* (Hook.) Meisn	Rutaceae	Shijiaocao	Luomechibozai	H	Whole plant	D	O	Cold, nephritis, pharyngitis	3	0.015
HXZ-505-31-09	*Heracleum repula* Franch	Apiaceae	Baiyunhuagen	Tongbonongcizai	H	Root	D	O	Asthma, cold, cough, lumbosacral pain	2	0.010
HXZ-506-1411-	*Ginkgo biloba* L	Ginkgoaceae	Yinxing	Niansantongmezai	T	Leaf	D	O	Angina pectoris	1	0.005
HXZ-51003-2	*Platycodon grandiflorus* A.DC	Campanulaceae	Jiegeng	Yanmepunizai	H	Root	D	O	Cough, pharyngitis	4	0.020
HXZ-51004-6	*Ipomoea cairica* (L.) Sweet	Convolvulaceae	Wuzhaojinlong	Minioumezai	HV	Root	D	O	Cough, edema	2	0.010
HXZ-51006-13	*Uncaria rhynchophylla* Miq	Rubiaceae	Gouteng	Aguzai	HV	Stem	D	O	Cold, hypertension	3	0.015
HXZ-51018-7	*Aster ageratoides* Turcz	Asteraceae	Sanmaiziwan	Laigumilongtongzai	H	Whole plant	D	O	Bronchitis, cold, pharyngitis	1	0.005
HXZ-51025-13	*Cymbopogon distans* (Nees ex Steud.) Will.Watson	Poaceae	Yunxiangcao	Sipanzai	H	Whole plant	D	O	Cold, diarrhea, rheumatism	1	0.005
HXZ-51107-1	*Elsholtzia bodinieri* Vaniot	Lamiaceae	Fengweicha	Longrinibaizai	H	Whole plant	D	O	Cold, dyspepsia, headache, hepatitis, pharyngitis, toothache	2	0.010
HXZ-51107-5	*Rhodobryum roseum* (Hedw.) Limpr	Bryaceae	Huixincao	Nimebunongcizai	B	Whole plant	D	O	Palpitation	3	0.015
HXZ-51107-9	*Viola japonica* Langsd. ex Ging	Violaceae	Litoucao	Zonggeidabosongzai	H	Whole plant	D	O	Eye disease, mastitis	1	0.005
HXZ-51114-1	*Lycium chinense* Mill	Solanaceae	Gouqi	Cinizai	S	Fruit	D	O	Lumbosacral pain, tinnitus	3	0.015
HXZ-51114-3	*Ophioglossum vulgatum* L	Ophioglossaceae	Pingerxiaocao	Hanmeluolianzai	H	Whole plant	D	O	Cough, eye disease	1	0.005
HXZ-41102-26	*Aralia elata* (Miq.) Seem	Araliaceae	Songmu	Zilaibumezai	T	Root	I	EA	Rheumatism, traumatic injury	1	0.005
HXZ-51115-2	*Bidens pilosa* L	Asteraceae	Guizhencao	Niannongwozai	H	Whole plant	D	O	Dysentery, pharyngitis	1	0.005
HXZ-51115-5	*Acorus calamus* L	Acoraceae	Shuichangpu	Rihamesizai	H	Rhizome	D	O	Epilepsy, stroke, tinnitus	1	0.005
HXZ-51129-7	*Cocculus orbiculatus* (L.) DC	Menispermaceae	Mufangji	Wobonongcizai	WV	Root	D	O	Eczema, edema, pharyngitis	2	0.010
HXZ-60911-3	*Agastache rugosa* Kuntze	Lamiaceae	Huoxiang	Alonganengzai	H	Whole plant	D	O	Cold, cough, dysentery, scabies	1	0.005
HXZ-60911-15	*Sanguisorba officinalis* L	Rosaceae	Diyu	Cimetongzai	H	Root	PW	EA	Burn and scald	1	0.005
HXZ-60911-24	*Sagittaria sagittifolia* L	Alismataceae	Cigu	Riabuzai	H	Corm	D	O	Stone	1	0.005
HXZ-60911-25	*Allium fistulosum* L	Amaryllidaceae	Cong	Acongtongzai	H	Whole plant	D	O	Cold	4	0.020
HXZ-60924-4	*Pachysandra axillaris* Franch	Buxaceae	Bandengguo	Sibadengsangazai	S	Whole plant	PU	EA	Rheumatism, traumatic injury	1	0.005
HXZ-41114-10	*Silene asclepiadea* Franch	Caryophyllaceae	Wacao	Gawuwuzai	H	Root	PU	EA	Rheumatism, traumatic injury	1	0.005
HXZ-50404-15	*Mucuna sempervirens* Hemsl	Fabaceae	Changchunyoumateng	Aniannujiezai	WV	Stem	D	O	Irregular menstruation, traumatic injury	1	0.005
HXZ-61002-6	*Biancaea sappan* (L.) Tod	Fabaceae	Sumu	Woguozizai	T	Branch	D	O	Dysmenorrhea, tetanus	1	0.005
HXZ-61003-5	*Gelsemium elegans* (Gardner & Champ.) Benth	Loganiaceae	Gouwen	Womanzengzizai	WV	Whole plant	PU	EA	Eczema, rheumatism	1	0.005
HXZ-61003-10	*Melicope pteleifolia* (Champ. ex Benth.) T.G.Hartley	Rutaceae	Sanyaku	Laigusanbuzai	T	Leaf	D	O	Eczema, hepatitis, pharyngitis	1	0.005
HXZ-61106-12	*Tripterygium hypoglaucum* Hutch	Celastraceae	Kunmingshanhaitang	Duosangulaiyuzai	S	Root	I	O	Fracture, rheumatism	3	0.015
HXZ-61106-13	*Forsythia suspensa* Vahl	Oleaceae	Lianqiao	Shanmishantongzai	S	Fruit	D	O	Cold	2	0.010
HXZ-61106-14	*Isatis tinctoria* L	Brassicaceae	Daqingye	Atongkaomezai	H	Leaf	D	O	Cold, parotitis, pharyngitis	1	0.005
HXZ-61106-20	*Dianthus superbus* L	Caryophyllaceae	Qumai	Shihuoluozai	H	Whole plant	D	O	Edema, eye disease	1	0.005
HXZ-61106-42	*Podophyllum versipelle* Hance	Berberidaceae	Bajiaolian	Niannongheitongzai	H	Leaf	PW	EA	Traumatic injury	2	0.010
HXZ-61113-3	*Dracaena cochinchinensis* (Lour.) S. C. Chen	Asparagaceae	Jianyelongxueshu	Tongsinizai	T	Leaf	D	O	Asthma, traumatic injury	3	0.015
HXZ-61113-4	*Oroxylum indicum* (L.) Kurz	Bignoniaceae	Muhudie	Quanduotongtouyuzai	T	Seed	D	O	Bronchitis, whooping cough	1	0.005
HXZ-61113-17	*Citrus limon* (L.) Osbeck	Rutaceae	Ningmeng	Sanganzimezai	S	Fruit	D	O	Cough, dyspepsia	2	0.010
HXZ-61119-6	*Phyllanthus puberus* (L.) Müll.Arg	Phyllanthaceae	Suanpanzi	Zhashuanduozai	S	Fruit	D	O	Malaria, pharyngitis, toothache	1	0.005
HXZ-61126-1	*Alstonia scholaris *(L.) R. Br	Apocynaceae	Tangjiaoshu	Wuyuduotongzai	T	Leaf	D	O	Asthma, bronchitis, malaria, whooping cough	1	0.005
HXZ-61126-11	*Mentha canadensis* L	Lamiaceae	Bohe	Caibinongmizai	H	Leaf	D	O	Cold, eczema, toothache	4	0.020
HXZ-61003-11	*Tinospora crispa* (L.) Hook.f. & Thomson	Menispermaceae	Qianlizhaogen	Jiechezai	HV	Stem	PU	EA	Snake bite, traumatic injury	1	0.005
HXZ-411-16-07	*Gaultheria leucocarpa *Blume	Ericaceae	Dianbaizhu	Nianzhazai	S	Whole plant	I	EA	Rheumatism, traumatic injury	1	0.005
HXZ-51122-4	*Dolichos trilobus* L	Fabaceae	Lianbiandou	Anudubuzai	HV	Whole plant	I	EA	Traumatic injury	3	0.015

Habit: H, Herb; S, Shrub; T, Tree; HV, Herbaceous vine; WV, Woody vine; B, Bryophyte

Preparation method: D, Decocted in water; PU, Pounded; PW, Powdered; I, Infused in liquor; Administration form: O, Oral; EW, External washing; EA, External application

**Fig. 4:**
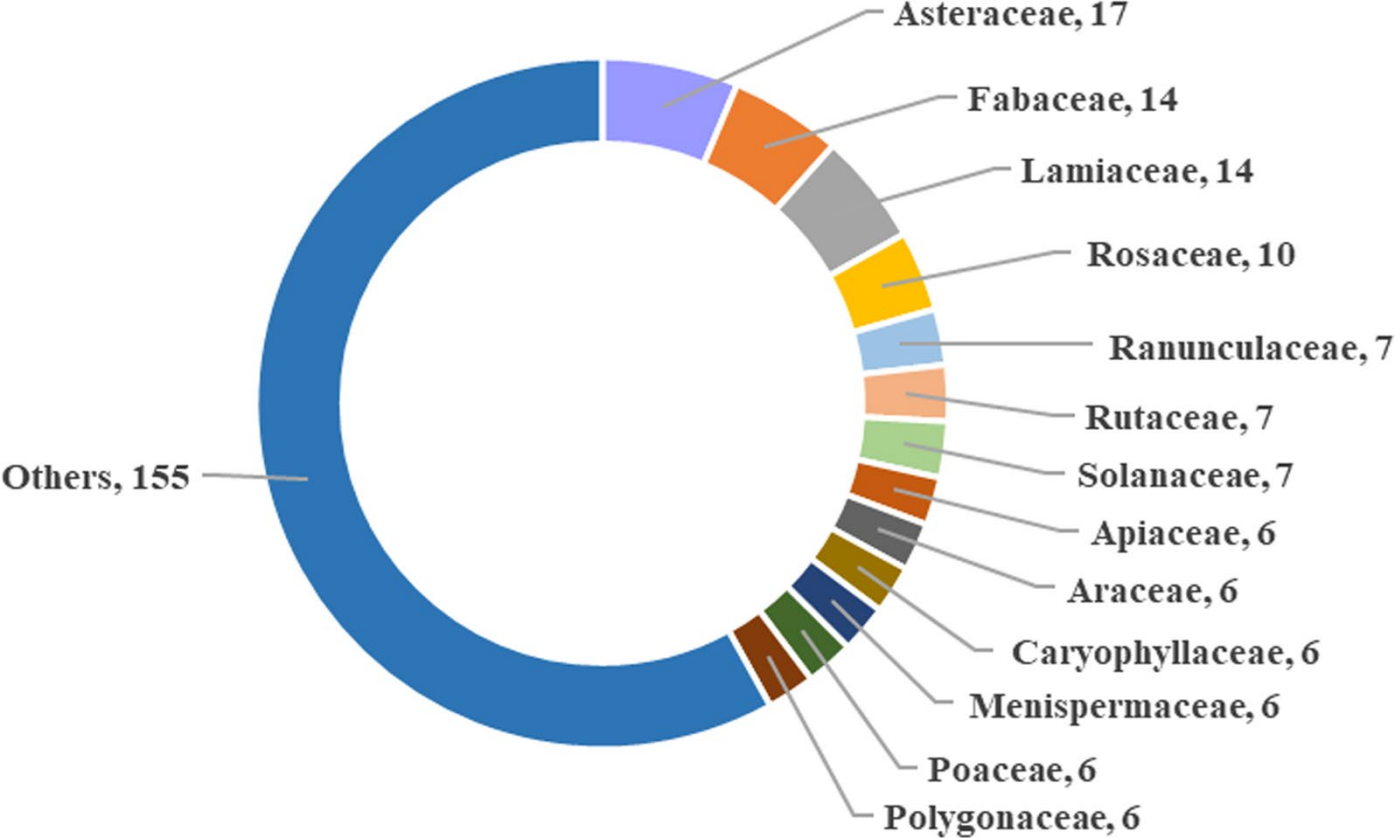
Family of investigated medicinal plants

The results of the statistical analysis of the plant families and species are shown in Fig. 4. Among the recorded medicinal plant species, most belonged to Asteraceae (17 species, 6.37%), Lamiaceae (14 species, 5.24%), Fabaceae (14 species, 5.24%), and Rosaceae (10 species, 3.75%). A previous study on the traditional market of Honghe Prefecture in Yunnan Province also reported that Asteraceae and Lamiaceae were the families that contained a significant number of medicinal plant species [27]. These four families accounted for 3.85% of the total number of families and 20.6% of the total number of species in the present study. This result revealed that local healers used a wide variety of medicinal plants from different families, but only a few families were highlighted. The remaining 155 species belonged to 91 families (1–5 species per family). The distribution of medicinal plant species within various families was relatively scattered, and local healers selected medicinal plants with a wide range to treat diseases, implying that they were proficient in using a variety of medicinal plants to treat various diseases. Although the medicinal plants used in the study area were diverse and rich, the availability of some herbs in local areas is becoming difficult because of overharvesting and agricultural development, such as *Paris polyphylla* var. *yunnanensis*, *Psammosilene tunicoides*, and *Tinospora sagittata* var. *yunnanensis*.

Nature worship of the Yi people has played a vital role in promoting the protection of the natural environment and enhancing the ecological consciousness of “harmony between man and nature.” For instance, the local Yi people believe that plants growing on the “Mi-zhi Mountain” are divine and must be protected and respected. Locals believed that those people who destroyed these plants would be punished by deities in the future. To ensure the authenticity of medicinal plants and the sustainability of resources, it is necessary to advocate the ecological concept of the local Yi people. Moreover, the local government could encourage research on the protection and propagation of wild species of Yi medicinal plants.

**Fig. 5:**
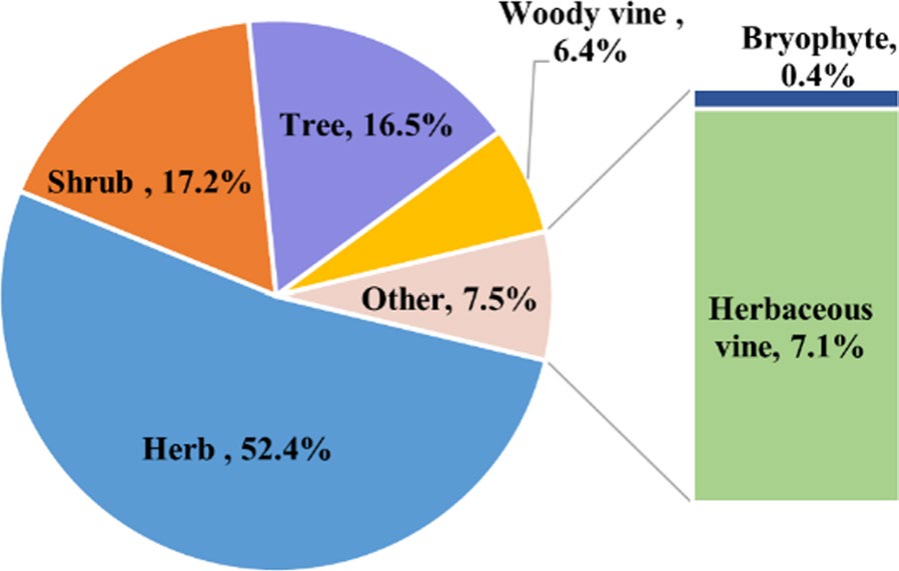
Life forms of medicinal plants in the study area

As depicted in Fig. 5, the medicinal plants observed in this study were classified into 140 species of herb (52.4%), 46 species of shrub (17.2%), 44 species of tree (16.5%), 19 species of herbaceous vine (7.1%), 17 species of woody vine (6.4%), and one species of bryophyte (0.4%). These results are consistent with those of other studies [28, 29]. Herbs and shrubs constituted a significant portion of the total species, which could be related to the local environment and human activities. From the environmental perspective, the study area is located within a typical karst area, so most plant life forms are herbaceous, followed by shrubs and trees, but vines are relatively uncommon. As far as human activities are concerned, herbaceous plants are easy to pick, cultivate, reproduce, and use. Furthermore, it was easier to collect shrubs because a variety of them were grown in the surrounding environment. Therefore, shrubs were the second most frequently used medicinal plants following herbs. Vine species were few in the study area, and most of them were grown in mountain forests; therefore, the process of collecting vines required more time and labor. Consequently, they were rarely used for medicinal purposes. The various medicinal plants used by local healers demonstrated that the local healers experimented with an extensive range of plants to treat diseases and accumulated abundant experience and unique knowledge of Yi medicine.

### Traditional uses and preparation of medicinal plants

In general, the effectiveness of medicinal plants depends heavily on the part of the plant used as medicine. Different medicinal parts of the same plant may have different efficacy levels. For instance, the root of *Panax notoginseng* is famous for its therapeutic effects, such as blood circulation improvement, blood stasis removal, and cardiovascular protection [30]. The flowers of *Panax notoginseng* have been reported to exhibit a variety of pharmacological activities for the treatment of hypertension, pharyngitis, and other inflammatory diseases because of the high content of saponins in it [31]. They are also widely used for the preparation of tea. The leaves of *Panax notoginseng* can be used to treat analgesia and inflammation. Therefore, to achieve the maximum therapeutic effect, it is crucial to select the appropriate medicinal part.

**Fig. 6:**
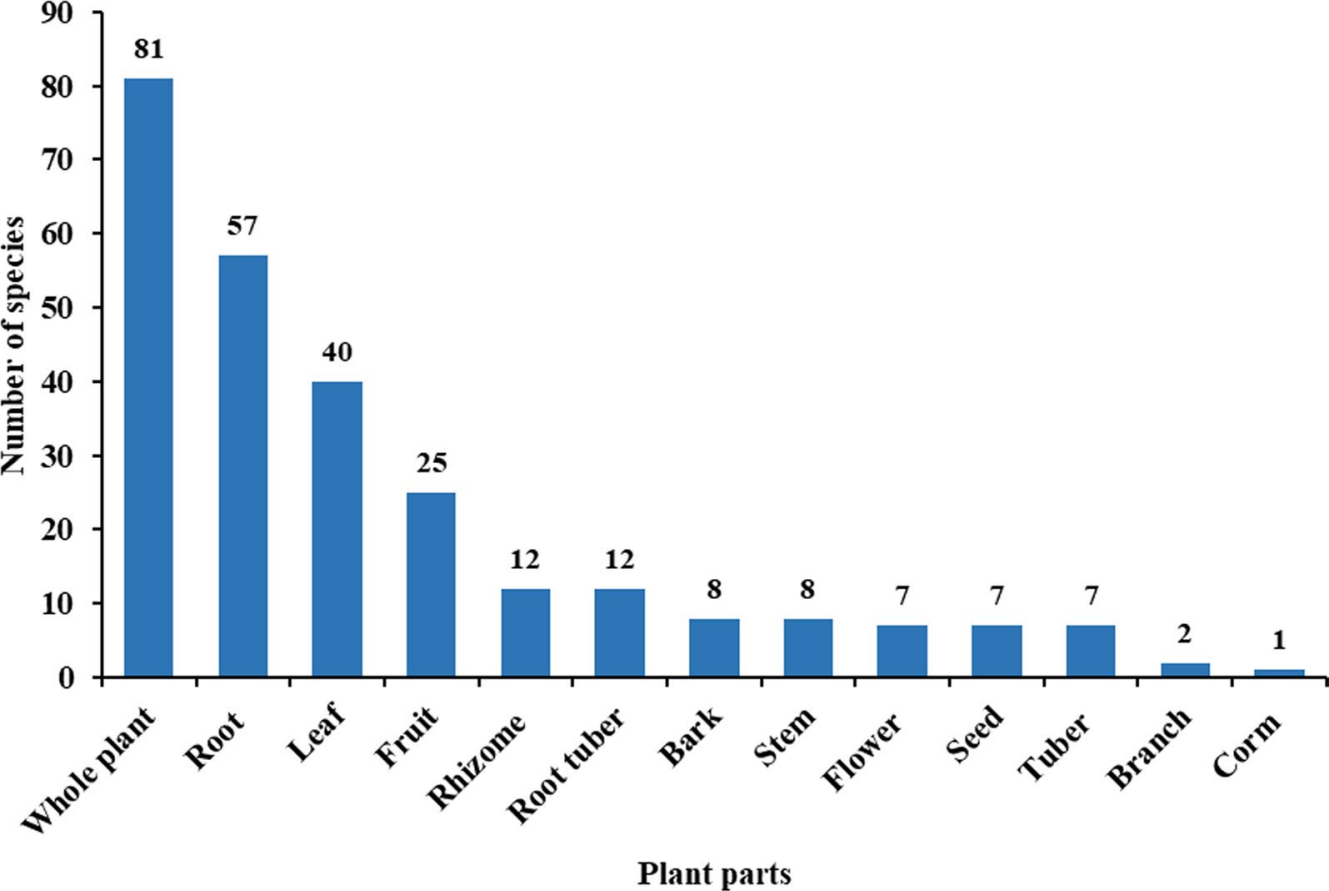
Parts of medicinal plants used by local healers in the study area

The informants described 13 categories of medicinal parts used to treat various diseases (Fig. 6). Among them, whole plants (81 species, 30.3%), roots (57 species, 21.3%), leaves (40 species, 15.0%), fruits (25 species, 9.4%), rhizomes (12 species, 4.5%), and root tubers (12 species, 4.5%) were the most commonly used parts by local healers. Although barks, stems, flowers, seeds, tubers, branches, and corms were also used, they accounted for only minor percentages. Similar ethnomedical practices have been reported in several other ethnobotanical investigations [32, 33].

**Fig. 7:**
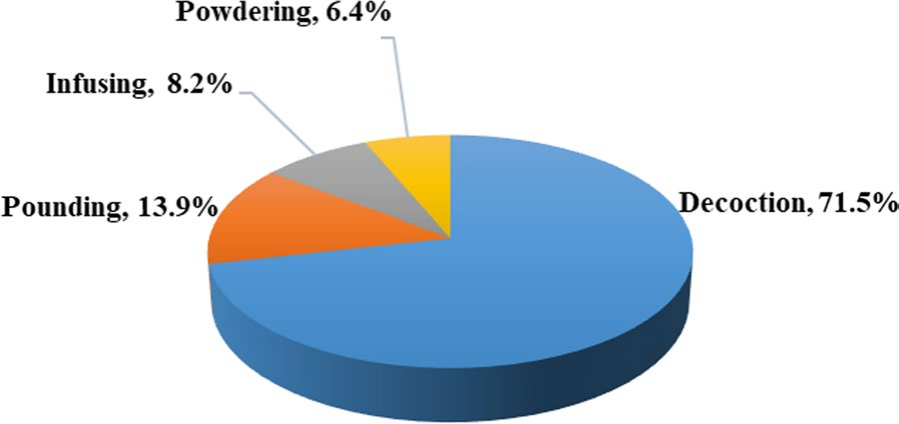
Preparation methods of herbal medicine

As illustrated in Fig. 7, traditional medicines were prepared by the Yi people in Mile through four primary methods, including decoction (71.5%), pounding (13.9%), infusing (8.2%), and powdering (6.4%). Decoction is generally considered by local healers to be the most common method of herbal medicine preparation because it can easily and effectively extract the active ingredients from the plant materials. Pounding is the next most commonly used method that can maintain the active components of fresh plants intact. Infusion with liquor is also commonly used by the locals for herbal preparation, which possesses the following three major advantages: (1) alcohol is an excellent solvent for extracting active components from herbs, (2) alcohol has antiseptic and antitoxic properties, which could delay hydrolysis and enhance the stability of several bioactive ingredients, and (3) easy preparation and convenient application. The Yi people believed that alcohol promotes blood circulation, which is especially beneficial for treating rheumatism and traumatic injuries. Therefore, they prepared the medicinal liquor by soaking medicinal plants in liquor for approximately 1 month in an airtight container. Numerous medicinal plants, such as *Dipsacus asper*, *Alangium chinense*, *Toddalia asiatica*, and *Aconitum vilmorinianum*, are often soaked in liquor by the locals for the treatment of traumatic injuries, fractures, and rheumatism. Based on the comparison of several studies, it is clear that the traditional Yi medicine used by the Yi people in Mile, Xiaoliangshan[23], and Chuxiong [13] areas are characterized by its use of liquor. This result may be closely related to Yi culture, in which liquor is adored by the Yi people and it is very important in their lives. In daily life, it is used for ceremonies and festivals, serviced to honored guests and friends, and used to prepare plant medicines as a common method.

**Fig. 8:**
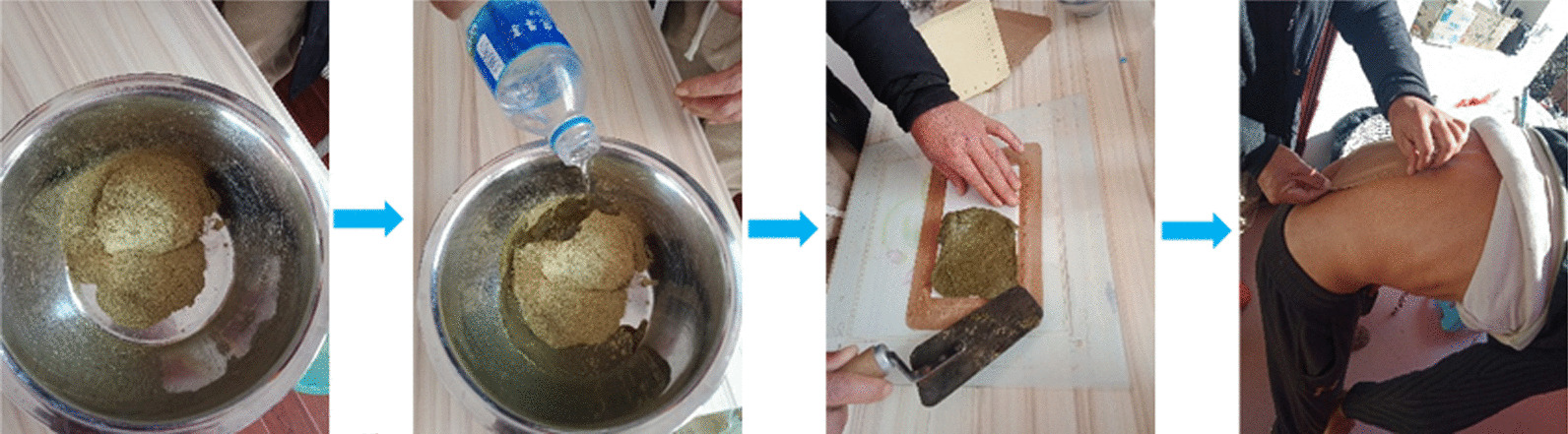
A patient with a strain of lumbar muscles is treated by a local healer. (The photos were taken by the author H.L., Photos were taken in January 2022.)

Oral administration possesses a series of advantages, such as high patient compliance, convenient administration, and minimal preparation. Similar to other studies [34, 35], oral administration was the most common form of administration in the present study. External administration was also preferred because of the toxicity of the plant and/or the disease specificity. Several external methods were commonly used by local healers, such as fresh herbs being directly mashed for external administration, dry herbs being ground into powder for external administration (Fig. 8), and herbs being decocted in water for bathing at the appropriate temperature. During medicinal baths, because the effective components of herbs can directly act on the infected or diseased region, their liver’s first pass effect and side effects to the stomach and intestine could be avoided. Moreover, herbal liquids with appropriate temperature could promote blood circulation, improve metabolism, and enhance the immune system.

In the study area, local healers incorporated medicinal baths with fumigation and steaming therapy to form a unique treatment known as “Sijuedu.” There are four primary steps in the drug therapy of “*Sijuedu*” ritual. First, the therapist rubs, pats, or bathes the patient with a herbal soup prepared using a variety of medicinal plants, such as *Dipsacus asper*, *Aconitum racemulosum*, and *Aconitum carmichaelii*. Second, the therapist places a hot stone slab on the floor next to the patient’s feet, and then the patient squats slightly to keep his/her body suspended above the hot slab. Third, the therapist pours the remaining medicinal soup over the hot stone slab to form steam. Finally, the therapist covers the patient’s body and the hot slab completely with a shawl so that the steam formed by the medicinal soup can effectively fumigate and steam the patient. This traditional remedy was considered a trusted indigenous medical practice by the locals because of its significant therapeutic effectiveness. This unique medical practice derived from the local unique philosophy, attitudes, beliefs, culture, and economic status was unknown publicly. Therefore, it is necessary to apply modern pharmacological methods and theories to investigate Yi traditional medicines and medicinal knowledge to improve public understanding and confidence in Yi traditional medicine.

### Informant consensus factor

A statistical analysis of ICF values was conducted to check the homogeneity of the information provided by local healers. The traditional medicinal plants documented in this study exerted excellent effects in treating 49 different diseases. Based on the information provided by the informants, the reported diseases were systematically categorized into 10 distinct groups (Table 3), including traumatic injury and orthopedic disorders, respiratory system diseases, immune system diseases, and digestive system diseases. It is well known that the occurrence of diseases is related to various factors, such as the local environment, climate, ethnic activities, and lifestyle. In the study area, the wet and cold living environment and the agriculture and forestry activities of the locals made them susceptible to rheumatism, traumatic injury, and fractures. Furthermore, the dry and windy weather in winter and spring increased dust particles in the air, thereby increasing the occurrence rate of respiratory diseases, such as cough, pharyngitis, and bronchitis, in the study area. Moreover, the locals easily catch a cold because of the dramatic temperature difference between day and night in mountainous regions. Digestive system disorders frequently occurred in the study area because of the preference for spicy and stimulating foods, irregular diets, and unclean living environments. Therefore, the locals accumulated abundant medicinal knowledge and experience for treating these diseases in long-term clinical practice.

**Table 3:** Informant consensus factor by categories in the study area

Category	Diseases	Number of diseases	Nur	Nt	ICF
Traumatic injury and orthopedic disorders	Traumatic injury (71), fracture (16), lumbosacral pain (18), muscle and bone pain (2)	4	107	16	0.86
Respiratory system diseases	Cold (34), pharyngitis (41), pneumonia (6), asthma (6), tuberculosis (2), whooping cough (4), parotitis (5), bronchitis (8), cough (22), rhinitis (1)	10	129	22	0.84
Immune system diseases	Rheumatism (81)	1	81	14	0.84
Digestive system diseases	Hepatitis (18), gastritis (4), dyspepsia (26), diarrhea (12), dysentery (16), ascaridiasis (3), constipation (9), stomachache (11)	8	99	20	0.81
Others	Cancer (2), edema (29), snake bite (17), malaria (5)	4	53	12	0.79
Nervous system diseases	Epilepsy (3), migraine (2), headache (11), tetanus (2)	4	18	6	0.71
Gynecological disorders	Irregular menstruation (20), metrorrhagia (6), mastitis (9), dysmenorrhea (11), menostasis (6)	5	52	15	0.73
Skin and facial diseases	Eczema (13), scabies (14), burn and scald (8), toothache (14), eye disease (6), tinnitus (7), dermatophytosis (2)	7	64	21	0.68
Circulation system diseases	Hypertension (5), angina pectoris (1), stroke (4), palpitation (3)	4	13	4	0.75
Urinary system diseases	Nephritis (9), stone (3)	2	12	5	0.64

In general, the higher the ICF value (closer to 1), the more diverse the plant species used by healers to treat a particular disease category [36], whereas the lower the ICF value (closer to 0), the more concentrated the plant species. In this study, ICF was calculated for each disease category, with the value ranging from 0.64 to 0.86. The highest ICF (0.86) was obtained from traumatic injury and orthopedic disorders, followed by respiratory system diseases (0.84), immune system diseases (0.84), and digestive system diseases (0.81). These four disease categories exhibited high ICF values (approximately 1), which could be related to the fact that local healers could obtain a variety of medicinal plants from wild habitats. A total of 107, 129, 81, and 99 plant species were collected and used by local healers to treat these four disease categories, respectively. Although abundant medicinal knowledge and experience were accumulated in this region, they were not widely shared among the locals because of the conservative inheritance of medicinal knowledge.

Urinary system diseases had the lowest ICF value (0.64). During long-term treatment, local healers had a high level of consensus on the medicinal plants they used. In this study, the local healers identified 12 medicinal plants as effective sources of treatment for these diseases.

### Relative frequency of citation

The RFC was calculated to determine the importance of medicinal plant species used by local healers to treat various diseases. Medicinal plants with high RFC values implied that these plants were widely used and well known among local people. This could be related to the positive therapeutic effect, abundance, and easy collection of these species. A total of 196 prescriptions were collected in this investigation, with the number of prescriptions mentioning a specific plant species (FC) ranging from 1 to 14. Among the 267 medicinal plants recorded, the FC values of 15 medicinal plant species were > 4, and their RFC values were 0.026–0.071 (Table 4). The RFC values of *Zingiber officinale*, *Lycopodium japonicum*, and *Aconitum carmichaelii* were higher. Further research on their chemistry, pharmacology, and toxicity is required to improve the development and utilization of these medicinal plants.

**Table 4:** Relative frequency of citation (RFC) of plant species mentioned in prescriptions (FC > 4)

Scientific name	FC	RFC
*Zingiber officinale*	14	0.071
*Lycopodium japonicum*	10	0.051
*Angelica sinensis*	7	0.036
*Aconitum carmichaelii*	7	0.036
*Dipsacus inermis*	6	0.031
*Panax notoginseng*	6	0.031
*Phryma leptostachya*	6	0.031
*Leonurus japonicus*	6	0.031
*Fagopyrum acutatum*	5	0.026
*Cyathula officinalis*	5	0.026
*Paris polyphylla* var*. yunnanensis*	5	0.026
*Ligusticum striatum*	5	0.026
*Taraxacum mongolicum*	5	0.026
*Plantago asiatica*	5	0.026
*Berberis bealei*	5	0.026

**Fig. 9:**
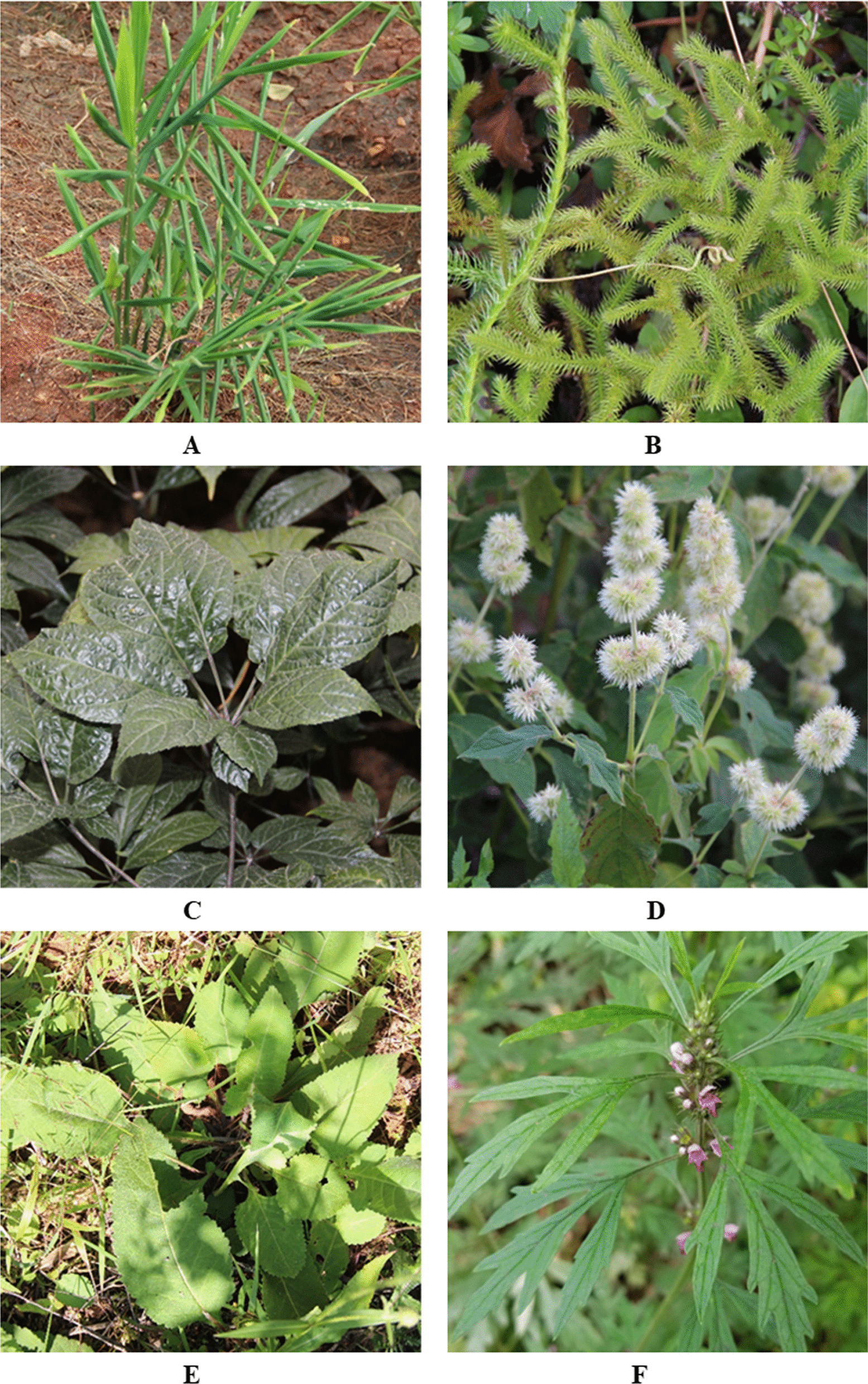
Selected medicinal plants in the study area **A*** Zingiber officinale,* **B** *Lycopodium japonicum*, **C** *Panax notoginseng*, **D** *Cyathula officinalis*, **E** *Dipsacus asper*, **F*** Leonurus japonicas *(The photos were taken by the author J.S., Z.B. and S.X., Photos were taken in May to August 2020.)

*Zingiber officinale* had the highest frequency of citation (FC = 14). It is a perennial herb belonging to the Zingiberaceae family (Fig. 9A) and is widespread throughout the study area. As a “medicinal food homology” plant, its fresh root was not only consumed as a spice but also used as a herbal medicine [37]. A recent study demonstrated that the root of *Zingiber officinale* contains > 300 chemical components, including various volatile oils, gingerol, and diarylheptanoids [38]. Modern pharmacological studies have reported that it exerts antioxidant, anti-inflammatory, antimicrobial, and anticancer effects [39]. In the study area, the local Yi people prepared a mixture of *Zingiber officinale* juice, honey, and boiling water in a setting ratio (1:2:3, v/v/v) and administered it orally to treat vomiting. A decoction of *Zingiber officinale* and *Allium fistulosum* was often used by the locals to cure colds. Furthermore, as a traditional remedy, *Zingiber officinale* slices were soaked in liquor by the locals for 3 days to treat skin diseases, such as tinea manuum, tinea pedis, and scabies. Sophisticated instruments can be used for the isolation and clear identification of more bioactive compounds in *Zingiber officinale*. Meanwhile, it is necessary to further investigate their biological activities and mechanisms of action.

Locals can easily collect *Lycopodium japonicum* (FC = 10) from the surrounding area because of the abundant resources in the study area (Fig. 9B). Previous studies have demonstrated that alkaloids and serratane-type triterpenoids are the typical constituents of *Lycopodium japonicum* [40, 41] and possess acetylcholinesterase inhibitory [42] and anti-inflammatory activities [43]. *Lycopodium japonicum*, *Cyathula officinalis*, and *Paederia foetida* were commonly soaked in liquor by local healers for 3–5 days to prepare a medicinal liquor used to treat rheumatoid arthritis and limb numbness by applying topically to the affected area.

The lateral root of *Aconitum carmichaelii* (FC = 7), known as “Fuzi” in Chinese medicine, is also an important Chinese medicinal material clinically used to treat rheumatoid arthritis, bronchitis, and pain in China, Japan, and other Asian regions [44]. More than 100 chemical compounds have been isolated from this medicinal plant, including alkaloids, flavonoids, and polysaccharides [45]. Modern pharmacological studies have demonstrated that aconitine, neaconitine, and other alkaloids exhibit effective anti-inflammatory and analgesic properties [46, 47]. *Aconitum carmichaelii* combined with other herbs can also exhibit significant anti-inflammatory activity for the treatment of related diseases. For instance, a previous study confirmed the in vivo and in vitro effects of *Fuzi Lizhong* decoction in treating nonalcoholic fatty liver disease [48].

*Aconitum carmichaelii* exhibits remarkable efficacy in treating pain and inflammation in the joints. Nonetheless, its use was limited due to its strong cardiotoxicity [49, 50]. Alkaloids derived from *Aconitum carmichaelii* have been demonstrated to be significant active and toxic components, damaging several organs such as the heart, liver, kidney, and nervous system. The safe and effective use of toxic herbs in clinical practice is extremely difficult without a clear understanding of the mechanisms of their toxicity. Nevertheless, the Yi people have accumulated rich experience in enhancing the efficacy and detoxifying the toxicity of herbal medicines in clinical practice and developed numerous processing methods, such as decocting for 45 min, soaking overnight with water in which rice has been washed, stir frying to yellow with wheat bran, stir frying with salt water, and stir frying with vinegar. It has been demonstrated that the addition of auxiliary substances could reduce the toxicity of herbal medicines [51]. The local Yi people used a similar method to reduce the toxicity of *Aconitum carmichaelii* by stewing it with pig feet. This medicinal diet is highly favored by the local people because it is delicious and healthy. During its preparation, several key steps must be paid more attention, viz. (1) fresh *Aconitum carmichaelii* and pig feet must be added to boiling water at the same time and continuously boiled for at least 24 h, (2) addition of boiling water is obligatory if it is necessary to add water to the pot during cooking, and (3) people should stay in a warm room for more than 8 h after consuming the medicinal diet and should not consume cold food or cold drinks.

### Fidelity level

We calculated the FL values for the 15 most preferred plant species to quantify their importance in treating a specific disease (Table 5). The FL values of these medicinal plants ranged from 26.09% to 80.77%. Higher FL values for medicinal plants indicate that these plants are essential in local settings, and healers are more likely to select these plants to treat a specific disease [52]. The FL values for four plants were > 70%, indicating that they played a critical role in treating the diseases mentioned in this study.

**Table 5:** Fidelity level of most frequently used plants for different ailment categories

Scientific name	Therapeutic uses	Ip	Iu	FL (%)
*Leonurus japonicus*	Irregular menstruation	21	26	80.77
*Panax notoginseng*	Traumatic injury	15	19	78.95
*Dipsacus asper*	Traumatic injury	12	16	75.00
*Cyathula officinalis*	Rheumatism	13	18	72.22
*Paris polyphylla* var.* yunnanensis*	Traumatic injury	14	21	66.67
*Lycopodium japonicum*	Traumatic injury	14	22	63.64
*Zingiber officinale*	Rheumatism	13	22	59.09
*Phryma leptostachya*	Scabies	4	7	57.14
*Plantago asiatica*	Eye disease	6	11	54.55
*Taraxacum mongolicum*	Hepatitis	8	15	53.33
*Ligusticum striatum*	Headache	5	10	50.00
*Fagopyrum acutatum*	Pharyngitis	10	21	47.62
*Angelica sinensis*	Rheumatism	5	11	45.45
*Aconitum carmichaelii*	Traumatic injury	9	22	40.91
*Mahonia bealei*	Hepatitis	6	23	26.09

After data analysis, the FL values for *Panax notoginseng* and *Paris polyphylla* var. *yunnanensis* used for treating traumatic injuries were 78.95% and 66.67%, respectively. *Panax notoginseng*, which has a high medicinal value, was widely used to prevent bleeding (Fig. 9C), enhance blood circulation, reduce swelling, relieve pain, enrich the blood, and maintain health [53]. The major active components of *Panax notoginseng* include saponins, polysaccharides, fatty acids, and flavonoids [54]. Previous studies have demonstrated that the saponins contained in *Panax notoginseng* are beneficial for preventing and treating cardiovascular diseases [55], cancer [56], and inflammatory response [57]. Although most pharmacological functions of this medicinal plant were primarily attributed to its saponin constituents [58], the other components of *Panax notoginseng* also exhibit significant hemostatic activity [59, 60]. Through deeper research, the more active ingredients of *Panax notoginseng* and their mechanism of action were gradually explored and clarified. Currently, a variety of therapeutic drugs and health care products derived from *Panax notoginseng* play a crucial role in maintaining people’s health. *Paris polyphylla* var*. yunnanensis* has also been effectively used for treating traumatic injuries in the clinic. Its rhizome is known as Chong-lou, which is widely used in traditional Chinese medicine and has been developed into a variety of commercially available products, such as “*Gongxuening Capsules*” and “*Yunnann Baiyao*” [61]. “*Yunnan Baiyao*” is widely used to treat traumatic injuries because of its significant hemostatic, anti-inflammatory, and analgesic properties. The high demand for *Panax notoginseng* and *Paris polyphylla* var. *yunnanensis* resulted in a decline of wild resources in natural habitats. To satisfy the needs of these two plants, their extensive cultivation was supported by the local government. Meanwhile, the management of these plants and the enhancement of fundamental research are also essential.

The FL values for *Cyathula officinalis* and *Dipsacus asper* used for treating rheumatism were 72.22% and 75.00%, respectively (Fig. 9D, E). *Cyathula officinalis* is a medicinal dietary plant commonly used as a tonic to nourish the liver and kidneys, strengthen bones and muscles, and promote blood flow in traditional healing practices [62]. In the study area, local healers commonly used its roots to treat bone injuries, osteoarthritis, and arthralgia. Modern pharmacological studies have reported that *Cyathula officinalis* roots exhibit antirheumatic, analgesic, and anti-inflammatory properties [63, 64], which supports the use of this plant in traditional medicine. *Dipsacus asper* is also an important medicinal plant for treating rheumatism. As a traditional Chinese medicinal plant containing triterpene glycosides, iridoid glycosides, phenolic acids, and volatile oil [65], *Dipsacus asper* is commonly used to treat osteoporosis, pain, fracture, and rheumatoid arthritis [66]. Furthermore, modern pharmacological studies have demonstrated that the compound Akebia saponin D derived from *Dipsacus asper* exhibits significant anti-inflammatory and analgesic activities by inhibiting the IL-6–STAT3–DNMT3b axis and activating the nuclear factor-E2-related factor 2 (Nrf2) signaling pathway [67]. *Cyathula officinalis* and *Dipsacus asper* possess significant potential as sources for the development of novel medicines for rheumatism.

*Leonurus japonicus* (80.77%) used for treating gynecological disorders also had a high FL value. Its aerial parts are widely used by local people to treat traumatic injuries (Fig. 9F), irregular menstruation, dysmenorrhea, amenorrhea, and other diseases. Because of its effective pharmacological activity against these gynecological diseases, *Leonurus japonicas* was also named “*Yi Mu Cao*” in China, which implies “a beneficial herb for mothers” [68]. To date, more than 130 chemical components have been isolated from *Leonurus japonicus* and identified, including alkaloids, diterpenes, flavones, phenylethanoid glycosides, and sesquiterpene glycosides [69, 70]. Pharmacological studies have demonstrated that several of these components exhibit good bioactivities, including antiplatelet aggregation, analgesia, anti-inflammation, neuroprotection, and anticancer activity [71, 72]. Folk medicine experiences and classical Chinese medicine records have indicated that *Leonurus japonicus* may be innocuous. However, recent toxicological studies have demonstrated that it exhibited some adverse effects and toxicity [73]. Therefore, to obtain the most benefit from *Leonurus japonicus*, different analysis methods must be applied to investigate its chemical constituents, pharmacological effects, and toxicological mechanisms.

The lowest FL value was obtained for *Mahonia bealei* (26.09%), which was used to treat hepatitis by local healers. Recent research has highlighted that *Mahonia bealei* possesses a variety of pharmacological activities, such as antibacterial, antioxidant, anti-inflammatory, and antitumor effects [74]. Alkaloids and phenolics were the major active constituents of this plant [75, 76]. Although numerous phytochemical and pharmacological studies have beene conducted, a systematic methodology has not been established to elucidate the molecular mechanisms underlying the antihepatitis activity of this plant. Therefore, further investigations are required. The low FL value for *Mahonia bealei* could be attributed to the fact that most informants were not aware of its dosage and preparation methods for hepatitis treatment.

### Comparison between Yi and other ethnic groups in China

In recent decades, ethnobotanical investigations of medicinal plant resources have attracted the attention of many ethnobotanical researchers and are becoming a research hotspot. In China, numerous ethnobotanical investigations of medicinal plants have focused on their traditional use by different ethnic groups, such as Dai, Tibetan, Yi, Yao, Shui, and Maonan. In this study, the medicinal plants used by the Yao [36], Shui [20], and Maonan [6] ethnic groups living in Gongcheng, Sandu, and Huanjiang, respectively, were selected to investigate the different uses of medicinal plants by the Yi people in Mile and other ethnic groups. A Venn diagram was used to visualize the medicinal plants used by the four ethnic groups.

**Fig. 10:**
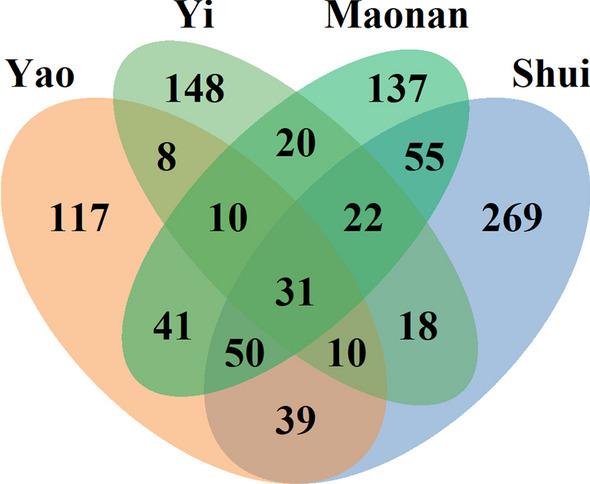
Comparison of medicinal plants between Yi and other ethnic groups in China

Before comparing the different uses of medicinal plants, it is crucial to understand the living environment of each ethnic group. The natural environment, means of livelihood, and the recorded medicinal plant species commonly used by the Yi, Yao, Shui, and Maonan groups are presented in Table 6. Obviously, these four locations are close to each other in terms of latitude and longitude and have similar natural environments, such as a subtropical monsoon climate and a mountainous region with complex terrain. Therefore, rich medicinal plant resources were nurtured and preserved in these four study areas. When we compared the medicinal plant species used by the Yi people in Mile with those used by the Yao, Shui, and Maonan people, we found that the number of overlapping medicinal plant species was 59, 53, and 83, respectively (Fig. 10). One medicinal plant could be easily found in different regions with similar natural environments, which could explain the crossover of medicinal plants between the Yi people and the Yao, Shui, and Maonan people, respectively.

**Table 6:** Study sites and characteristics of their natural environment

Ethnic groups	Study areas	Locations	Topography	Climate	Means of livelihood	Recorded medicinal plants
Yi	Mile City (Yunnan Province)	23° 50′–24° 39′ N 103° 04′–103° 49′ E	Mountainous, karst landform	Subtropical monsoon climate	Agriculture and forestry	267 species
Yao	Gongcheng County (Guangxi Province)	24° 37′–25° 17′ N 110° 36′–111° 10′ E	Mountainous, karst landform	Subtropical monsoon climate	Agriculture	306 species
Shui	Sandu County (Guizhou Province)	25° 10′–25° 30′ N 107°40′–108° 14′ E	Mountainous	Subtropical humid monsoon climate	Agriculture and forestry	505 species
Maonan	Huanjiang County (Guangxi Province)	24° 83′–25° 06′ N 107°92′–108° 26′ E	Mountainous	Subtropical monsoon climate	Agriculture and forestry	368 species

Disease incidence is often closely related to the local environment, climate, and lifestyle. As shown in Table 6, the people in these four places are primarily engaged in agriculture and forestry for their livelihood. The local people live and work in mountainous areas with environmental conditions such as dense forests, rainy weather, wind, and a large temperature difference, which are conducive to the development of rheumatism, traumatic injury, cold, and cough. Therefore, 31 species of medicinal plants were used by all four ethnic groups (Fig. 10), and many of them were used to treat rheumatism, traumatic injury, cold, and cough. The local people have accumulated a great deal of experience in treating these diseases.

From the perspective of traditional medicine development in these regions, the Yi, Yao, Maonan, and Shui ethnic groups all share a similar medical development history. For instance, in remote mountainous areas and regions with poor economic conditions, traditional remedies using medicinal plants were the most important or the only source of therapeutics available because modern medicine was not widely available. Consequently, to prevent and treat various diseases, these ethnic groups have developed their own medical knowledge systems with unique characteristics. Ethnic groups with different traditional cultures may use the same medicinal plant to treat different diseases. Therefore, although there were 31 species of overlapping medicinal plants, their use was not the same completely. For instance, *Coix lacryma-jobi* L. is commonly used by the Yi people to treat dyspepsia in Mile, whereas the Maonan people use it to treat acute nephritis in Huanjiang County. It is also used by the Yao people living in Gongcheng County to treat other diseases, such as infertility, rheumatism, stone formation, bad urination and defecation, hemorrhoids, and moist heat.

As depicted in Fig. 10, there are 148 medicinal plants commonly used by the Yi people in Mile that do not overlap with those used by the Yao, Shui, and Maonan people. Regarding the geographical environment, the Yunnan Province borders the Guizhou Province and Guangxi Province, but the ethnic minorities living in these three provinces have few opportunities for communication and learning of medical knowledge because of the mountains, inconvenient transportation, and poor information. Therefore, the medicinal plants used tend to have their own characteristics, with fewer crossovers.

## Conclusion

Mile is known for its abundance of medicinal plants and diverse national cultures, and the Yi people living in this area have a long history of using medicinal plants to treat various diseases in their daily lives. The data in this study were collected from 114 informants distributed in 5 townships in Mile. A total of 267 medicinal plant species belonging to 232 genera and 104 families were commonly utilized by the local Yi people. Our results confirmed that medicinal plants used by the Yi people in Mile are extremely diverse. Roots and whole plants of medicinal plants were commonly used by Yi healers in the form of decoction. The Yi communities have abundant traditional medical knowledge and are skilled at using unique remedies to ensure treatments are more convenient and effective as well as exhibit distinctive regional characteristics. The medicinal plants recorded in this study were used by local healers to treat 49 diseases, and a considerable number of them were used to treat respiratory diseases, rheumatism, traumatic injury, fractures, and digestive system diseases, which were frequent in the study area. Quantitative analyses (RFC and FL) were conducted to evaluate the importance of medicinal plants commonly used by locals. Plants such as *Zingiber officinale*, *Lycopodium japonicum*, *Aconitum carmichaelii*, *Panax notoginseng*, *Cyathula officinalis*, *Leonurus japonicus* played crucial roles in disease prevention and treatment.

Despite the abundant medicinal resources and knowledge in the study area, the inheritance of this valuable culture is facing serious challenges, including the decreasing number of local healers, aging of healers, lack of successors, and loss of traditional Yi medical knowledge. Moreover, environmental changes and overexploitation of wild resources are increasingly causing a lack of wild resources. Therefore, to better protect, inherit, and use local traditional medicinal knowledge and plant resources, it is extremely urgent to protect cultural diversity, perform systematic research on traditional Yi medicinal plant knowledge, promote the research and development of local characteristic wild plant resources, and establish a special base for medicinal materials. Meanwhile, the concept of “harmony between man and nature” in traditional Yi culture should be advocated and effectively integrated into modern development to better protect and utilize local traditional medicinal plant resources.

In conclusion, this ethnobotanical survey provides a useful reference for understanding the rich ethnobotanical knowledge of the Yi people. This survey also emphasizes the need to conduct further research to explore the therapeutic properties of these medicinal plants and protect the traditional Yi medicinal knowledge.

## Data Availability

All data generated or analyzed during this study are included in this published article.

## Abbreviations

ICF: Informant consensus factor
RFC: Relative frequency of citation
FL: Fidelity level
